# Ranked Subtree Prune and Regraft

**DOI:** 10.1007/s11538-023-01244-2

**Published:** 2024-01-31

**Authors:** Lena Collienne, Chris Whidden, Alex Gavryushkin

**Affiliations:** 1 Biological Data Science Laboratory, School of Mathematics and Statistics, University of Canterbury, Christchurch, New Zealand; 2 Faculty of Computer Science, Dalhousie University, Halifax, Canada; 3 Biomathematics Research Centre, University of Canterbury, Christchurch, New Zealand

**Keywords:** Phylogenetics, Time trees, Tree Rearrangements, Ranked trees, Treespace, Tree distance

## Abstract

Phylogenetic trees are a mathematical formalisation of evolutionary histories between organisms, species, genes, cancer cells, etc. For many applications, e.g. when analysing virus transmission trees or cancer evolution, (phylogenetic) time trees are of interest, where branch lengths represent times. Computational methods for reconstructing time trees from (typically molecular) sequence data, for example Bayesian phylogenetic inference using Markov Chain Monte Carlo (MCMC) methods, rely on algorithms that sample the treespace. They employ tree rearrangement operations such as $\textrm{SPR}$ (Subtree Prune and Regraft) and $\textrm{NNI}$ (Nearest Neighbour Interchange) or, in the case of time tree inference, versions of these that take times of internal nodes into account. While the classic $\textrm{SPR}$ tree rearrangement is well-studied, its variants for time trees are less understood, limiting comparative analysis for time tree methods. In this paper we consider a modification of the classical $\textrm{SPR}$ rearrangement on the space of ranked phylogenetic trees, which are trees equipped with a ranking of all internal nodes. This modification results in two novel treespaces, which we propose to study. We begin this study by discussing algorithmic properties of these treespaces, focusing on those relating to the complexity of computing distances under the ranked $\textrm{SPR}$ operations as well as similarities and differences to known tree rearrangement based treespaces. Surprisingly, we show the counterintuitive result that adding leaves to trees can actually decrease their ranked $\textrm{SPR}$ distance, which may have an impact on the results of time tree sampling algorithms given uncertain “rogue taxa”.

## Introduction

Phylogenetic trees are used to display evolutionary relationships, for example between organisms, species, or genes, and are usually inferred from DNA, RNA, amino acid, or other types of sequence data. Typical applications include reconstructing the evolutionary history of a set of species, e.g. to construct the tree of life (Hug 2016), analysing transmission patterns of viruses (Rambaut et al. 2001; Turakhia 2022), and investigating cancer evolution (Alves et al. 2019). The goal is the same in all applications: finding a phylogenetic tree that best explains evolutionary relationship between given (sequence) data, represented by the leaves of the tree.

Many methods for inferring phylogenetic trees from sequence data use tree sampling or treespace search algorithms. These include Bayesian methods, for example the software packages BEAST (Drummond and Rambaut 2007), BEAST2 (Bouckaert et al. 2014), MrBayes (Huelsenbeck and Ronquist 2001), and RevBayes (Höhna et al. 2016), and maximum likelihood methods like IQ-TREE (Nguyen et al. 2015), PhyML (Guindon et al. 2010), or RAxML (Stamatakis 2014). All these methods rely on tree proposals: for a given tree, a new tree similar to the current tree is proposed and is accepted if it fulfils certain conditions. If accepted, the current tree is updated to be this new tree and the procedure repeated. Tree rearrangement operations, which apply local changes to a tree, are commonly used for these tree proposals. Most popular are the Subtree Prune and Regraft ($\textrm{SPR}$) and Nearest Neighbour Interchange ($\textrm{NNI}$) tree rearrangements, the latter being a version of $\textrm{SPR}$ restricted to being more local.

An $\textrm{SPR}$ operation (or move) cuts an edge of a phylogenetic tree and reattaches the thereby detached subtree at a different position in the tree. This tree rearrangement (or a version of it) is implemented in many tree inference software packages, including maximum likelihood methods (Guindon et al. 2010; Stamatakis 2014; Price et al. 2010), Bayesian inference methods (Drummond and Rambaut 2007; Höhna et al. 2016; Ogilvie et al. 2017) and also recent parsimony-based methods that are able to infer large-scale phylogenies (Ye et al. 2022). One reason for the popularity of $\textrm{SPR}$ moves in tree search algorithms is that, unlike very local $\textrm{NNI}$ moves, $\textrm{SPR}$ moves can be used to jump across wider regions of treespace, preventing tree search algorithms from getting stuck in local optima (Guindon et al. 2010; Nguyen et al. 2015). Similarly, it has been shown for Bayesian inference methods that $\textrm{SPR}$ moves, or modifications thereof, can speed up convergence of Markov chains and improve their mixing when used in combination with other operators (Höhna et al. 2008). There is however a major obstacle for interpreting how well treespace is traversed when using $\textrm{SPR}$ moves for tree search and tree sampling algorithms: computing the $\textrm{SPR}$ distance, i.e. the minimum number of $\textrm{SPR}$ moves required to transform one tree into another, is $\mathcal{N}\mathcal{P}$-hard (Hickey et al. 2008; Bordewich and Semple 2005). Despite this $\mathcal{N}\mathcal{P}$-hardness result, fixed-parameter tractable algorithms for computing $\textrm{SPR}$ distances exist (Whidden et al. 2013), which can be used for analysing modes of posterior distributions (Whidden and Matsen 2015) or for computing supertrees (Whidden et al. 2013; Whidden and Matsen 2019). Understanding properties of the $\textrm{SPR}$ treespace, which can be viewed as a graph where vertices represent trees that are connected by edges if the trees are connected by an $\textrm{SPR}$ operation, has proven useful for getting a better understanding of phylogenetic inference methods (Whidden and Matsen 2019).

Analysing the geometry of the classic $\textrm{SPR}$ treespace has lead to numerous results for $\textrm{SPR}$ on rooted and unrooted trees (Whidden and Matsen 2017, 2019). Although $\textrm{SPR}$ operations are defined in the same way for both types of trees and the two resulting treespaces share most of their properties, the techniques to prove those properties differ significantly. $\mathcal{N}\mathcal{P}$-hardness of the problem of computing distances, for example, has first been shown for the rooted $\textrm{SPR}$ (Bordewich and Semple 2005) and only three years later for the unrooted case (Hickey et al. 2008). An important mathematical structure for both of these proofs is the Maximum Agreement Forest (MAF). A MAF for two trees results from deleting a minimum number of edges in each tree so that the two resulting graphs (forests) are isomorphic. The number of connected components of a MAF for two rooted trees coincides with their rooted $\textrm{SPR}$ distance (Bordewich and Semple 2005). For unrooted trees, however, this relationship between the $\textrm{SPR}$ distance and the number of connected components of the MAF does not hold (Allen and Steel 2001; Whidden and Matsen 2019), which was the reason some erroneous proofs of $\mathcal{N}\mathcal{P}$-hardness made it to the literature. It also has been found that the maximum distance under both rooted and unrooted $\textrm{SPR}$ is linear in the number of leaves (Ding et al. 2011; Atkins and McDiarmid 2019), while the number of trees in the 1-neighbourhood, i.e. the number of trees resulting from one $\textrm{SPR}$ move, is quadratic in the number of leaves (Song 2003; Allen and Steel 2001). The latter is an important property that has been used to determine the curvature of the treespace by Whidden and Matsen (2017) in order to analyse mixing properties of Markov Chain Monte Carlo methods, which are used for Bayesian tree inference.

The majority of these results have been derived for trees that do not contain timing information of evolutionary events. We refer to trees where branch lengths represent times, meaning that the evolutionary events represented by internal nodes are dated, as time trees. These time trees are of interest in many applications. Software packages like BEAST (Drummond and Rambaut 2007) and BEAST2 (Bouckaert et al. 2014), for example, infer time trees and rely on tree proposals that incorporate timing information of evolutionary events. A version of $\textrm{SPR}$ moves for time trees has been introduced by Höhna et al. (2008). The authors analysed the suitability of this move as a tree proposal operator for Bayesian inference using MCMC algorithms and showed it performed better than some of the previous operators in isolation but it was still better to use a combination. As of May 2023, this move is default in BEAST.^1^ A guided version of this proposal was introduced in Höhna and Drummond (2012), where guiding the choice of destination improved the acceptance ratio. Another version of $\textrm{SPR}$ moves, which works simultaneously on a species tree and multiple gene trees, can be found in the Stacey package for BEAST2 (Jones 2017). Even though $\textrm{SPR}$ moves are widely used as tree proposals and have been adapted to work for time trees, little research has gone into $\textrm{SPR}$ treespaces that take times of evolutionary events into account.

A version of $\textrm{SPR}$ moves for time trees that has been studied mathematically is the one introduced by Song (2006), where ranked trees are considered. Ranked trees (which are called ordered trees in Song (2006)) are rooted phylogenetic trees with internal nodes ordered according to times of corresponding evolutionary events and leaves are assumed to be sampled at the same time (ultrametric). The $\textrm{SPR}$ moves defined by Song (2006) can move a subtree to a different place in the tree under the condition that the rank of the reattachment node is greater than the rank of the root of the moved subtree. Bounds for neighbourhood sizes and diameters are provided (Song 2006), but no further results are known. Despite these efforts to investigate $\textrm{SPR}$ moves for ranked trees, there is still a gap for analysing tree inference methods using $\textrm{SPR}$ for time trees, as the version of $\textrm{SPR}$ moves as defined by Song (2006) is currently not used in phylogenetic inference.

In this paper we fill this gap by considering an alternative definition of $\textrm{SPR}$ moves for ranked trees, where we require the height (i.e. rank) at which a subtree is cut and reattached to be the same. This restriction on the rank reattachment is inspired by ranked $\textrm{SPR}$ moves in phylogenetic inference software: The Fixed Node Prune and Regraft move introduced in Höhna et al. (2008) as tree proposal for Bayesian inference methods is the extension of our ranked $\textrm{SPR}$ moves to time trees. We call these tree rearrangements Horizontal $\textrm{SPR}$ ($\textrm{HSPR}$) moves and the corresponding treespace $\textrm{HSPR}$. Motivated by the importance of this move in computational phylogenetics, we study mathematical properties of this treespace focusing on the metric space of ranked trees that is given by the $\textrm{HSPR}$ move. Studying this treespace helps us understand its fundamental properties, which is important to analyse tree inference algorithms using $\textrm{HSPR}$ for tree proposals as well as posterior distributions of time trees output by BEAST or BEAST2, similar to how it has been done by Whidden and Matsen (2015). By additionally allowing rank moves, which swap the order of two nodes in a ranked tree, we define a further metric space called $\textrm{RSPR}$ (Ranked $\textrm{SPR}$). We include rank moves in the same way as it has been done in a variation of $\textrm{NNI}$ introduced in Gavryushkin et al. (2018) for ranked trees ($\textrm{RNNI}$) that allows distances to be computed in polynomial time (Collienne and Gavryushkin 2021), unlike in the classical $\textrm{NNI}$ treespace, where this problem is $\mathcal{N}\mathcal{P}$-hard. This suggests that the complexity of computing distances can be different in classical $\textrm{SPR}$ and its ranked version.

This paper is structured as follows. We introduce notations and define the new treespaces in Sect. 2 before discussing some of its fundamental properties in Sect. 3. This especially includes the cluster property, which a treespace possesses if shortest paths between trees preserve shared information in the form of common clusters. This property has proven to be important for the polynomial time algorithm in $\textrm{RNNI}$ (Collienne and Gavryushkin 2021) and for fixed-parameter tractable algorithms in $\textrm{SPR}$ (Whidden and Matsen 2019; Linz and Semple 2011). The discussion of the cluster property is followed by some observations on the shape of shortest paths and the relationship of $\textrm{RSPR}$ and $\textrm{HSPR}$ shortest paths (Sect. 4). Finally, we establish a surprising result on how the distance between two trees can change after adding a new leaf to them (Sect. 5). We provide an open source implementation of horizontal $\textrm{SPR}$ moves (Collienne 2023), which we use to show some properties of ranked $\textrm{SPR}$ spaces computationally.

## Preliminaries

**Fig. 1:**
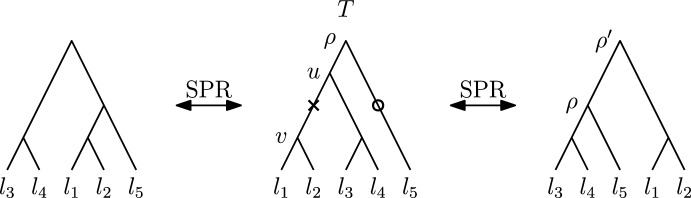
Two rooted $\textrm{SPR}$ moves. The cross marks the edge that is cut to prune the subtree $T|_v$ of *T* with leaf set $\{l_1, l_2\}$. In the tree on the left this subtree is reattached on the edge highlighted by a circle, connecting $l_5$ with its parent, and in the tree on the right the pruned subtree is reattached as child of a newly introduced root $\rho '$

A *rooted binary phylogenetic tree* is a pair $T = (t, \phi )$ where *t* is a rooted binary tree and $\phi : L(t) \rightarrow X$ is a bijective map from the set of leaves *L*(*t*) of *t* to a set of labels $X = \{l_1, l_2, \dots , l_n\}$. Throughout this paper, we refer to rooted binary phylogenetic trees simply as *rooted trees*, and we assume that all trees have *n* leaves, unless stated otherwise. Let *T* be a rooted tree that contains an edge $e = (u,v)$ and let $T|_v$ be the subtree of *T* rooted in *v*. A *subtree prune and regraft* ($\textrm{SPR}$) move on *e* in *T* transforms this tree into a new rooted binary rooted phylogenetic trees by the following three steps:

(i) *Prune the subtree* $T|_v$: delete the edge *e* from *T*, resulting in two connected components $T|_v$ and $T_\rho$, where $T_\rho$ contains the root $\rho$ of *T*.
(ii) Suppress the resulting node of degree two in $T_\rho$ (node *u*), so that $T_\rho$ is a rooted tree.
(iii) *Reattach* $T|_v$: either by introducing a new node *w* on an edge *f* in $T_\rho$ and adding an edge (*w*, *v*), or by adding a new root $\rho '$ and adding edges $(\rho ', \rho )$ and $(\rho ', v)$ (Fig. 1).

### Theorem 1

The decision problem $\textrm{SPR}$:

- Instance: Two rooted trees *T* and *R* and an integer *k*
- Question: Is $$d_{\textrm{SPR}}(T,R) \le k$$?

is $$\mathcal{N}\mathcal{P}$$-complete.

A proof for this theorem can be found in Bordewich and Semple (2005). This proof relies on the equivalence of the $\textrm{SPR}$ distance between two trees and the size of a maximum agreement forest. A *maximum agreement forest* (MAF) of two rooted trees *T* and *R* can be interpreted as a forest that results from cutting the minimum number of edges from both *T* and *R* to result in the same forest, and its size $|\textrm{MAF}(T,R)|$ is defined as the number of its connected components. For a formal definition, see Bordewich and Semple (2005). The equality $|\textrm{MAF}(T,R)| = d_{\textrm{SPR}}(T,R)$ implies that the same edge is only pruned once on a shortest $\textrm{SPR}$ path. Furthermore, distance computation can be broken down into smaller problems if two trees share some information in the form of common clusters (Linz and Semple 2011), which we will formally define later.

### Ranked Trees

A *ranked binary phylogenetic tree* is a pair $T = (T_u, \textrm{rank})$ consisting of a rooted tree $T_u$ and a function $rank:V \rightarrow \{0, 1, \dots , n-1\}$, where *V* is the set of nodes of $T_u$, such that: (i) $\textrm{rank}(v) = 0$ if and only if *v* is a leaf, (ii) $\textrm{rank}(v) \ne rank(w)$ for all internal nodes $v \ne w$, and (iii) $\textrm{rank}(v) < \textrm{rank}(w)$ if *w* is on the path from the root of $T_u$ to *v*. We refer to $\textrm{rank}(v)$ as the *rank of* *v*, and we denote node of rank *i* in a ranked binary phylogenetic tree *T* by $(T)_i$. For simplicity of notation, we say *tree* or *ranked tree* to refer to ranked binary phylogenetic trees, unless stated otherwise. The assumption that all leaves have rank 0 can be interpreted as requiring ranked trees to be ultrametric.

**Fig. 2:**
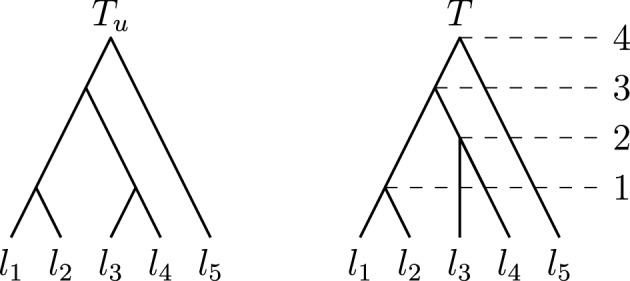
A rooted tree $T_u$ on the left and a ranked tree $T = (T_u, \textrm{rank})$ on the right

If $T = (T_u, \textrm{rank})$ is a tree, we call the rooted tree $T_u$ the *unranked version* of *T*, as it can be interpreted as *T* without ranks, but with leaf labels. Two trees *T* and *R* are *identical* if there is a graph isomorphism between them that preserves leaf labels and ranks. We then write $T \simeq R$, and if the trees are not identical we write $T \not \simeq R$. An example of a tree *T* with annotated ranks and its unranked version $T_u$ is given in Fig. 2.

A *subtree* $T|_v$ of a tree *T* is a tree rooted in a node *v* of *T* that contains exactly the nodes that descend from *v* in *T*, annotated by the same ranks as in *T*. We then say that $T|_v$ is *induced* by the node *v*. Note that by this definition of subtrees, the subtree of a ranked tree is not necessarily a ranked tree itself. We denote the parent of a node *v* by $\textrm{parent}(v)$ and if a subtree $T|_v$ is induced by *v*, we call $\textrm{parent}(v)$ *parent of the subtree* $T|_v$. To emphasise that we are considering the parent of a subtree $T|_v$ in a tree *T* we might write $\textrm{parent}_T(T|_v)$.

Since we consider trees where internal nodes are assigned unique ranks and leaves are assigned unique leaf labels, we can uniquely identify nodes in a tree by their labels, using the label function

1 $$\begin{aligned} l(v) = {\left\{ \begin{array}{ll} \textrm{rank}(v) &{} \quad \text { if } v \text { is internal node} \\ \phi (v) &{} \quad \text { if } v \text { is leaf}. \end{array}\right. } \end{aligned}$$

Throughout this paper we assume $\{1, \dots , n-1\} \cap \{l_1, \dots , l_n\} = \emptyset$, which results in *l* being a bijective function. We can therefore uniquely identify a node *v* by its label *l*(*v*), so we will refer to a node *v* simply by *l*(*v*). For example, we might refer to the internal node with rank *i* as *node* *i* and to the leaf with label $l_j$ as *node* $l_j$ or *leaf* $l_j$. We define a strictly partial order $\prec _T$ on the co-domain of *l* so that $l(u) \prec _T l(v)$ if $\textrm{rank}_T(u) < \textrm{rank}_T(v)$. If it is clear that we consider the tree *T* we might simply write $\prec$ for $\prec _T$.

Using the label function (1) allows us to represent edges (*u*, *v*) in a tree as (*l*(*u*), *l*(*v*)). We call the set $E(T) = \{(l(v), l(w))\ |\ (v,w) \text { is an edge in the tree } T\}$ the *edge set of* *T*. *E*(*T*) uniquely defines *T*. We say that an edge $(l^+,l^-)$ *covers* a rank $i \in \{1, \dots , n-1\}$ if $l^- \prec i \prec l^+$.

A *cluster* *C* of a tree *T* is the set of leaves descending from an internal node *v* in *T*. We then say that the node *v* *induces* the cluster *C*. A *cherry* is a subtree consisting of one internal node with two leaves as children, and we refer to a cherry by its cluster containing these two leaves, e.g. $\{c_1, c_2\}$. If the internal node of such a cherry has rank *i*, we say that the cherry $\{c_1, c_2\}$ has rank *i*. Given a set of leaves *S* and a tree *T*, the subtree $T|_S$ of *T* *induced by* *S* is the subtree of *T* with minimum number of leaves that contains all leaves of *S*. If *S* is a cluster of *T*, $T|_S$ contains exactly the leaves of *S*.

**Fig. 3:**
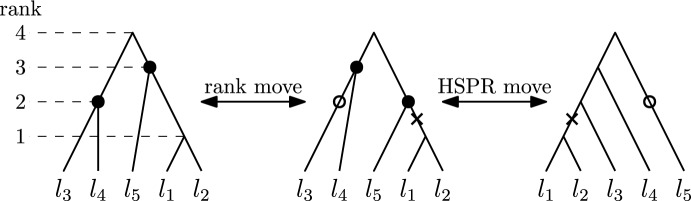
Rank move on the left, swapping the ranks of the nodes highlighted the leftmost tree. On the right an $\textrm{HSPR}$ move at rank 2 is illustrated, moving the subtree induced by $\{l_1,l_2\}$ by cutting the edge highlighted by a cross and re-attaching it at the edge highlighted by a circle

We can uniquely represent a tree *T* by a list of its clusters sorted according to increasing rank. This representation is called *cluster representation* and has been introduced by Collienne and Gavryushkin (2021). The leftmost tree in Fig. 3 for example has cluster representation $[\{l_1, l_2\}, \{l_3, l_4\}, \{l_1, l_2, l_5\}, \{l_1, l_2, l_3, l_4, l_5\}]$. We include the cluster induced by the root in the cluster representation of a tree, even though this cluster is simply the set of all leaf labels $\{l_1, l_2, \dots , l_n\}$ for all trees on *n* leaves.

### Subtree Prune and Regraft for Ranked Trees

A *horizontal* $\textrm{SPR}$ *move* ($\textrm{HSPR}$ move) on an edge *e* of a tree *T*, which cannot be incident to the root, transforms this tree into a tree $T'$ by shifting the top node of *e* horizontally to another branch. Formally, the move is performed in the following three steps:

(i) *prune the subtree* $T|_v$: delete the edge *e* to obtain two subtrees $T|_v$ and $T_\rho$, where $T_\rho$ has the same root $\rho$ as *T*,
(ii) suppress the resulting node of degree 2 in $T_\rho$ (top node of edge *e*), whose rank we denote by *i*,
(iii) *reattach* $T|_v$ *on an edge* *f*: reattach the root of $T|_v$ to a newly introduced node *v* at rank *i* on an edge *f* that covers rank *r* in $T_\rho$.

Since the changes done to the tree *T* move node *i*, we call this an $\textrm{HSPR}$ *move at rank* *i*. A *rank move* on a tree *T* can be applied to two nodes with consecutive ranks if they are not connected by an edge, and swaps the ranks of these two nodes. An example of an $\textrm{HSPR}$ move and a rank move can be found in Fig. 3.

Note that $\textrm{HSPR}$ moves and rank moves are reversible and in contrast to $\textrm{SPR}$ moves for rooted trees, $\textrm{HSPR}$ moves do not allow subtrees to be reattached above the root of a tree.

We are now ready to introduce our main objects of study in this paper, two treespaces extending the classical SPR treespace to ranked trees.

The Ranked $\textrm{SPR}$ $(\textrm{RSPR})$ space is a graph where vertices represent trees on *n* leaves that are connected by an edge if one tree can be transformed into the other by an $\textrm{HSPR}$ move or a rank move. The Horizontal $\textrm{SPR}$ $(\textrm{HSPR})$ space is a graph where vertices represent trees on *n* leaves that are connected by an edge if one can be transformed into the other by an $\textrm{HSPR}$ move.

We refer to the moves allowed in $\textrm{RSPR}$ space, i.e. rank moves and $\textrm{HSPR}$ moves, as $\textrm{RSPR}$ *moves*.

A *path* between two trees in $\textrm{HSPR}$ ($\textrm{RSPR}$) is a sequence of trees $p = [T_0, T_1, \dots , T_d]$ such that $T_i$ and $T_{i+1}$ are connected by an $\textrm{HSPR}$ ($\textrm{RSPR}$) move for all *i*. If a path *p* contains $d+1$ trees, we say it has *length* $|p| = d$. A *shortest path* between trees *T* and *R* is a path of minimal length connecting *T* and *R*. The length of such a path is called the *distance* between trees *T* and *R*, and we refer to this distance as $d_{\textrm{HSPR}}(T,R)$ in $\textrm{HSPR}$ space and $d_{\textrm{RSPR}}(T,R)$ in $\textrm{RSPR}$ space.

#### $\textrm{HSPR}$ moves on edge sets

Let us consider how an $\textrm{HSPR}$ move changes the set of edges of a tree. By the definition of $\textrm{HSPR}$ moves, there are four edges involved in an $\textrm{HSPR}$ move at rank *i* on a tree *T*: The three edges incident to the node of rank *i*, and the edge *f* on which the pruned subtree gets reattached. All other edges stay the same in the tree *T* and its $\textrm{HSPR}$ neighbour $T'$. Let $e = (i, l)$ be the edge that is cut by the $\textrm{HSPR}$ move between *T* and $T'$, let $(i^+, i)$ and $(i, i^-)$ be the other two edges incident to *i*, and let $f = (j^+, j^-)$ be the reattachment edge in *T*. Then the $\textrm{HSPR}$ move between *T* and $T'$ changes these edges as follows:

(i) *prune the subtree* $T|_v$: delete the edge $e = (i,l)$
(ii) suppress the resulting node of degree 2: replace edges $(i^+, i)$ and $(i, i^-)$ by an edge $(i^+, i^-)$
(iii) *reattach* $T|_v$: replace edge $f = (j^+, j^-)$ by $(j^+, i)$ and $(i, j^-)$ and add edge (*i*, *l*).

**Fig. 4:**
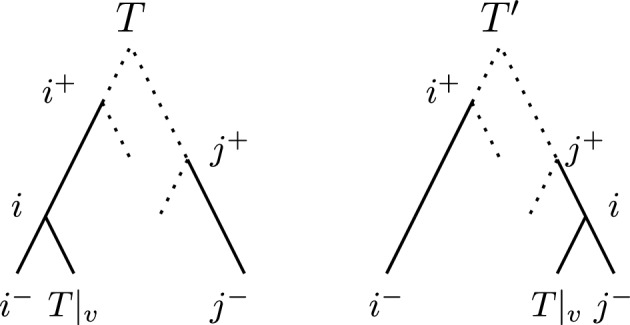
$\textrm{HSPR}$ move pruning the subtree $T|_v$, moving it to the edge $(i^+, i^-)$. Dotted edges represent parts of the tree that potentially contain further nodes

An illustration of the trees *T* and $T'$ with these node labels is provided in Fig. 4. The difference between the edge sets of *T* and $T'$ can be summarised to

$$\begin{aligned} E(T') = E(T)&\setminus \{(i^+, i), (i, i^-), (j^+, j^-)\} \\&\cup \{(i^+, i^-), (j^+, i), (i, j^-)\}. \end{aligned}$$

Conversely, if an edge set $E(T')$ can be described in this way, then *T* and $T'$ are connected by an $\textrm{HSPR}$ move:

##### Theorem 2

Let *E*(*T*) be the set of edges of a tree *T*. A tree $T'$ with

$$\begin{aligned} E(T') = E(T)&\setminus \{(i^+, i), (i, i^-), (j^+, j^-)\} \\&\cup \{(i^+, i^-), (j^+, i), (i, j^-)\} \end{aligned}$$

is $\textrm{HSPR}$ neighbour of *T* if and only if $(i^+, i), (i, i^-), (j^+, j^-) \in E(T)$ and $j^- \prec i \prec j^+$.

Note that $j^- \prec i \prec j^+$ is required in Theorem 2, as the reattachment edge needs to cover the rank *i* of the $\textrm{HSPR}$ move.

Using Theorem 2, we can define an $\textrm{HSPR}$ move between trees *T* and $T'$ by providing the difference in edge sets *E*(*T*) and $E(T')$. We say that a change in edge sets describes a *valid* $\textrm{HSPR}$ move, if the edges that are removed from *E*(*T*) fulfil the conditions of Theorem 2.

#### $\textrm{HSPR}$ moves on cluster representation

Similar to using edge sets to describe $\textrm{HSPR}$ moves, we can also use the cluster representation to describe an $\textrm{HSPR}$ move between two trees. Let *T* and *R* be trees connected by an $\textrm{HSPR}$ move at rank *i* as described in Theorem 2 and let the clusters induced by $i^-$, $T|_v$ (the subtree that is moved), and $j^-$ be *A*, *B*, and *C*, respectively. Any of *A*, *B*, and *C* could be just a leaf, e.g. $A = \{l_k\}$ for some *k*, but for simplicity we refer to those sets as clusters, too. Let furthermore $T = [C_1, C_2, \dots , C_{i-1}, A \cup B, C_{i+1}, \dots , C_{n-1}]$ be the cluster representation of *T*. The $\textrm{HSPR}$ move described in Theorem 2 then creates a new cluster $B \cup C$ at rank *i*, as in $T'$ the node *i* has $T|_v$ and $j^-$ as children. All clusters induced by nodes with rank less than *i* remain unchanged between *T* and $T'$. Since the subtree induced by *B* becomes sibling of the subtree induced by *C* in $T'$, the move between *T* and $T'$ removes *B* from every cluster of *T* that contains *B* but not *C* and is induced by a node with rank greater than *i*. On the other side, *B* is added to every cluster induced by a node with rank greater than *i* that contains *C* in *T*. All remaining clusters induced by nodes with rank greater than *i* that do not contain *B* or *C* remain unchanged between *T* and $T'$.

We can summarise this to describe $T'$ by its cluster representation

$$\begin{aligned} T' = [C_1, \dots , C_{i-1}, B \cup C, C'_{i+1}, \dots , C'_{n-1}] \end{aligned}$$

with

$$\begin{aligned} C'_m = {\left\{ \begin{array}{ll} C_m \setminus B &{} \quad \text { if } B \subset C_m \text { and } C \not \subset C_m \\ C_m \cup B &{} \quad \text { if } C \subset C_m \\ C_m &{} \quad \text { otherwise}. \end{array}\right. } \end{aligned}$$

Conversely, if the difference between the cluster representation of *T* and $T'$ can be described in this way, *T* and $T'$ are connected by an $\textrm{HSPR}$ move:

##### Theorem 3

Let $T = [C_1, C_2, \dots , C_{i-1}, A \cup B, C_{i+1}, \dots , C_{n-1}]$ be a tree and $C = C_l$ for some $l \in \{1, \dots , i-1\}$. A tree

$$\begin{aligned} T' = [C_1, \dots , C_{i-1}, B \cup C, C'_{i+1}, \dots , C'_{n-1}] \end{aligned}$$

is connected to *T* by an $\textrm{HSPR}$ move if and only if

$$\begin{aligned} C'_m = {\left\{ \begin{array}{ll} C_m \setminus B &{} \quad \text { if } B \subset C_m \text { and } C \not \subset C_m \\ C_m \cup B &{} \quad \text { if } C \subset C_m \\ C_m &{} \quad \text { otherwise}. \end{array}\right. } \end{aligned}$$

## Basic Properties of $\textrm{HSPR}$ and $\textrm{RSPR}$

The first question we want to answer is whether our newly defined treespaces are connected (Theorem 4), that is, whether a tree can be transformed into any other tree by a sequence of moves in $\textrm{HSPR}$ or $\textrm{RSPR}$. Connectedness is essential for these treespaces, as tree rearrangements are used for tree proposals in (MCMC) random walks, which should be able to reach any tree in treespace from any starting tree. For developing and interpreting such random walks it is furthermore important to know how many trees have distance one from a given tree, as well as the maximum distance between any two trees. We establish neighbourhood size (Theorem 5) and maximum distance (diameter) in $\textrm{RSPR}$ and $\textrm{HSPR}$ in Sect. 3.1. We then investigate the cluster property for $\textrm{RSPR}$ and $\textrm{HSPR}$ and explain the significance of neither of the two spaces having this property (Sect. 3.2).

### Theorem 4

The treespaces $\textrm{HSPR}$ and $\textrm{RSPR}$ are connected.

### Proof

We show that any pair of trees $T'$ and *R* are connected by a path of only $\textrm{HSPR}$ moves. As all $\textrm{HSPR}$ moves are $\textrm{RSPR}$ moves, it then follows that both spaces are connected.

**Fig. 5:**
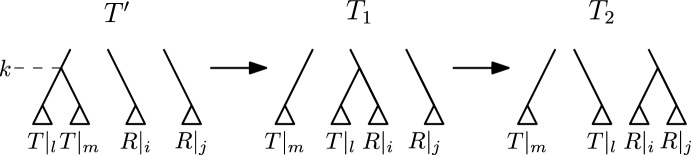
Path between *T* and $T_2$ as described in the proof of Theorem 4. Only subtrees involved in the moves given in the theorem are shown in this illustration

We construct an $\textrm{HSPR}$ path from $T'$ to *R* by the following bottom up approach, iterating through ranks $k = 1, \dots , n-1$ of *R*. In every iteration *k*, we perform $\textrm{HSPR}$ moves so that all nodes with ranks less than or equal to *k* induce the same clusters in the tree after iteration *k* and *R*. Let *T* be the tree before iteration *k*, let $R|_i$ and $R|_j$ be the subtrees that are children of the node of rank *k* in *R*, and let $T|_l$ and $T|_m$ be the subtrees that are children of the node of rank *k* in *T*, i.e. $\textrm{parent}_{R}(R|_i) = \textrm{parent}_{R}(R|_j) = k$ and $\textrm{parent}_{T}(T|_l) = \textrm{parent}_{T}(T|_m) = k$. We can assume that $R|_i$ and $R|_j$ are subtrees in *T*, because we use a bottom up approach that results in all nodes of rank less than *k* inducing the same cluster in *R* and the tree *T* before iteration *k*. Note that in the first iteration $k=1$, $R|_i$ and $R|_j$ will contain a single leaf only. Consider the following path transforming *T* into a tree $T_2$ with $\textrm{parent}_{T_2}(R|_i) = \textrm{parent}_{T_2}(R|_j) = k$ (see Fig. 5):

First, perform an $\textrm{HSPR}$ move at rank *k* that prunes $T|_l$ from *T* and reattaches it on the edge between $R|_i$ and $\textrm{parent}_{T}(R|_i)$. In the resulting tree $T_1$, $\textrm{parent}_{T_1}(R|_i) = \textrm{parent}_{T_1}(T|_l) = k$. In a second step, we perform an $\textrm{HSPR}$ move pruning the subtree $R|_i$ from $T_1$ and reattaching it on the edge between $R|_j$ and $\textrm{parent}_{T_1}(R|_j)$, resulting in a tree $T_2$ with $\textrm{parent}_{T_2}(R|_i) = \textrm{parent}_{T_2}(R|_j) = k$. Since all moves between *T* and $T_2$ are $\textrm{HSPR}$ moves at rank *k*, no clusters induced by nodes less than *k* change between *T* and $T_2$, while the cluster induced by the node of rank *k* changes so that it coincides between $T_2$ and *R*. Hence, all cluster induced by nodes with rank less than or equal to *k* are identical in $T_2$ and *R*. Note that if $\{R|_i,R|_j\}$ and $\{T|_l,T|_m\}$ intersect, the trees $T_1$ or $T_2$ might be equal to *T*, and fewer than two steps are required to reach $T_2$. At the end of iteration *k*, we update $T := T_2$ and continue with the next iteration $k+1$. After iteration $n-2$, we reach *R*, as after each iteration *k* all nodes with rank less than or equal to *k* induce the same clusters in *T* and *R*.

This procedure can be applied to any two trees to compute a path connecting them by a sequence of $\textrm{HSPR}$ moves, which proves the theorem. $\square$

The algorithm used to prove Theorem 4 produces a path between any two trees in $\textrm{HSPR}$, and can hence be used to approximate $\textrm{HSPR}$ distances. An implementation can be found on GitHub (Collienne 2023).

Since $\textrm{HSPR}$ and $\textrm{RSPR}$ are connected undirected graphs, we obtain the following corollary.

### Corollary 1

$d_{\textrm{HSPR}}$ and $d_{\textrm{RSPR}}$ are metrics.

The *(1-)neighbourhood* of a tree *T* in a treespace with distance measure *d* is defined as $NH(T):=\{T'\ |\ d(T,T')=1\}$ and a tree $T' \in NH(T)$ is called *neighbour* of *T*. We use $NH_{\textrm{HSPR}}(T)$ and $NH_{\textrm{RSPR}}(T)$ to refer to the neighbourhood of *T* in $\textrm{HSPR}$ and $\textrm{RSPR}$, respectively. Because tree inference algorithms often require sampling tree neighbourhoods, it is important to know the number of *neighbours* of a tree *T* under a tree rearrangement, i.e. |*NH*(*T*)|. In rooted and unrooted $\textrm{SPR}$ the number of neighbours of a tree is quadratic in the number of leaves *n* (Song 2003; Allen and Steel 2001).

For counting the number of neighbours of a tree in $\textrm{RSPR}$, we need the following notion: If a tree *T* has two nodes *r* and $r+1$ with rank difference one that are not connected by an edge, we say that $[r+1, r]$ is a *rank interval*. The leftmost tree *T* in Fig. 3 for example has two rank intervals: [3, 2] and [2, 1]. We now show that the number of neighbours in $\textrm{RSPR}$ and $\textrm{HSPR}$ is quadratic in the number of leaves, with the number of neighbours in $\textrm{RSPR}$ naturally depending on the shape of the tree. We derive both of these numbers explicitly.

### Theorem 5

The number of neighbours of a tree *T* with *k* rank intervals is $|NH_{\textrm{RSPR}}(T)| = (n-1)(n-2) + k$ in $\textrm{RSPR}$ and $|NH_{\textrm{HSPR}}(T)| = (n-1)(n-2)$ in $\textrm{HSPR}$.

### Proof

The number of neighbouring trees resulting from a rank move on a tree with *k* rank intervals is *k*, as there is one unique rank move for every such interval.

We now count the number of $\textrm{HSPR}$ moves at rank *i* for $i \in \{1, \dots , n-1\}$. Since every node has two children, two different subtrees can be pruned by an $\textrm{HSPR}$ move at rank *i*. There are $n-1-i$ edges that cover rank *i*, excluding the one on which the node of rank *i* is placed in *T*, so there are $n-1-i$ potential reattachment edges for the pruned subtree. This gives $2 (n-1-i)$ $\textrm{RSPR}$ neighbours of *T* resulting from an $\textrm{HSPR}$ move at rank *i*. And because $\textrm{HSPR}$ moves can be performed at any rank between 1 and $n-1$, the number of $\textrm{RSPR}$ moves possible on *T* is:

$$\begin{aligned} 2 \sum \limits _{i=1}^{n-1}(n-1-i) = (n-1)(n-2) \end{aligned}$$

Since all rank moves and $\textrm{HSPR}$ moves result in different trees, *T* has $(n-1)(n-2)$ neighbours in $\textrm{HSPR}$ and $(n-1)(n-2)+k$ neighbours in $\textrm{RSPR}$. $\square$

### Diameter

The maximum distance between any two trees in a treespace with distance measure *d*, $\max \limits _{T,R} d(T,R)$, is called the *diameter* of the treespace. When measuring the similarity of trees using a distance metric, knowing the maximum possible distance is essential for interpreting distances. The diameter of both unrooted and rooted $\textrm{SPR}$ space is $n - \Theta (\sqrt{n})$ (Ding et al. 2011; Atkins and McDiarmid 2019) and hence linear in *n*. We show in this section that the diameters of $\textrm{HSPR}$ space and $\textrm{RSPR}$ space are linear in the number of leaves *n*, too, by establishing lower and upper bounds.

#### Corollary 2

The diameters of $\textrm{HSPR}$ space and $\textrm{RSPR}$ space have an upper bound of ${2(n-2)}$.

#### Proof

We can use the algorithm introduced in the proof of Theorem 4 to compute a path between any two trees *T* and *R* in $\textrm{HSPR}$. Since every $\textrm{HSPR}$ move is an $\textrm{RSPR}$ move, the length of this path is an upper bound of the diameter of both $\textrm{HSPR}$ and $\textrm{RSPR}$ space. The algorithm uses a bottom-up approach that, starting at tree *T*, constructs in iteration $k= 1, \dots , n-2$ the cluster induced by the node of rank *k* in *R*, using at most two $\textrm{HSPR}$ moves. After iteration $n-2$, we receive the destination tree *R* after at most $2(n-2)$ $\textrm{HSPR}$ moves. Because the path *p* constructed by this algorithm has length at most $2 (n-2)$, this provides an upper bound to the diameter of $\textrm{HSPR}$ and $\textrm{RSPR}$. $\square$

It is important to note that the algorithm described in the proof of Theorem 4 approximates $\textrm{HSPR}$ distances and does not compute the exact distance for all pairs of trees, which we can show using our implementations (Collienne 2023).

We can also prove a lower bound for the distance between any two trees in $\textrm{HSPR}$, but first we need the following lemma.

#### Lemma 1

Let *T* and *R* be trees containing *x* leaves whose parents have different ranks in *T* and *R*, i.e. $|\{(u,v)\ |\ v \text { is leaf and } (u,v) \in E(T) \text { and } (u,v) \notin E(R)\}| = x$. Then $d_{\textrm{HSPR}}(T,R) \ge \lceil \frac{x}{2}\rceil$.

#### Proof

As described in the technical introduction, an $\textrm{HSPR}$ move at rank *i* between trees *T* and $T'$ leads to the following difference in edge sets for some edges $(i^+, i), (i, i^-), (j^+, j^-) \in E(T)$ where $(j^+, j^-)$ covers rank *i*:

$$\begin{aligned} E(T') = E(T) \setminus \{(i^+,i), (i,i^-), (j^+, j^-)\} \cup \{(i^+, i^-), (j^+, i), (i, j^-)\}. \end{aligned}$$

Therefore, the only nodes whose parents change by this $\textrm{HSPR}$ move are the nodes *i*, $i^-$, and $j^-$. Not all of them need to have different a parent after the move, since for example $i^+ = j^+$ results in the parent of *i* having the same rank in *T* and $T'$. With $(i^+,i)$ and $(i,i^-)$ being edges in *T*, it follows that *i* is an internal node, so only $i^-$ and $j^-$ can be leaves. Therefore, an $\textrm{HSPR}$ move can change the parents of at most two leaves.

Since the ranks of *x* parents of leaves differ between *T* and *R* and any $\textrm{HSPR}$ move can fix at most two of those, there are in total at least $\lceil \frac{x}{2} \rceil$ $\textrm{HSPR}$ moves needed to connect *T* and *R*. $\square$

**Fig. 6:**
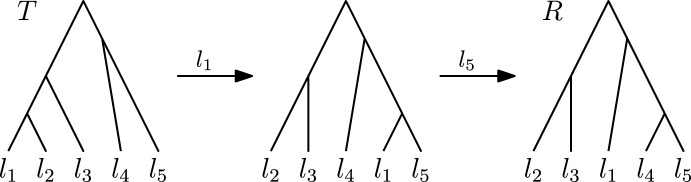
Trees *T* and *R* of the counterexample to the weak cluster property in $\textrm{RSPR}$ and $\textrm{HSPR}$ in Theorem 7. The labels of the arrows indicate leaves that are pruned in the corresponding $\textrm{HSPR}$ moves

The lower bound given in Lemma 1 can be tight. For example, the two leftmost trees in Fig. 6 are connected by one $\textrm{HSPR}$ move and the parents of $x=2$ leaves ($l_2$ and $l_5$) have different ranks in the two trees.

#### Theorem 6

There are trees *T* and *R* with distance $d_{\textrm{HSPR}}(T,R) \ge \lceil \frac{n}{2} \rceil$.

#### Proof

Let *T* and *R* be the following caterpillar trees:

$$\begin{aligned} T&= [\{l_1, l_2\},\{l_1, l_2, l_3\}, \{l_1, l_2, l_3, l_4\}, \dots , \{l_1, l_2, \dots , l_n\}] \\ R&= [\{l_3, l_4\},\{l_2, l_3, l_4\}, \{l_2, l_3, l_4, l_5\}, \dots , \{l_2, \dots , l_n\}, \{l_1, \dots , l_n\}] \end{aligned}$$

The parent of $l_1$ and $l_2$ has rank one in *T*, but not in *R*, where $l_3$ and $l_4$ are children of the node of rank one. For all other leaves $l_i$ with $i \in \{5, \dots , n\}$, the rank of the parent also is different in *T* than in *R*: $\textrm{parent}_T(l_i) = i - 1 \ne i - 2 = \textrm{parent}_R(l_i)$. Therefore, the parents of all *n* leaves have different ranks in *T* and *R*, and by Lemma 1 it follows $d_{\textrm{HSPR}}(T,R) \ge \lceil \frac{n}{2} \rceil$. $\square$

By Corollary 2 and Theorem 6, the diameter of the $\textrm{HSPR}$ space is linear in *n*. We will see later (Corollary 6) that Theorem 6 also applies to $\textrm{RSPR}$.

### No Cluster property

Two trees *T* and *R* *share* a cluster *C* if both of them contain *C* as cluster. If on a path *p* every tree contains the cluster *C*, we say that *p* *preserves* the cluster *C*. We moreover say that a treespace has the *strong cluster property* (simply called *cluster property* by Collienne et al. (2021)) if for two trees sharing a cluster *C*, every shortest path between them preserves *C*. In other words, if two trees share some evolutionary information in form of a cluster, this information is preserved along every shortest path between them if the treespace has the cluster property. If for any two trees sharing a cluster *C* there exists a shortest path that preserves *C* in a tree space, we say that this treespace has the *weak cluster property*. Note that the difference between the weak and the strong cluster property is that for the strong cluster property we require all shortest paths to preserve clusters, while for the weak cluster property only one shortest path needs to preserve a shared cluster.

The classic rooted (unranked) version of $\textrm{SPR}$ space has the weak cluster property, which is shown as part of the proof of Theorem 2.2 in Linz and Semple (2011), where the problem of computing the $\textrm{SPR}$ distance is split into the problem of computing distances for subtrees induced by shared clusters. The weak cluster property of $\textrm{SPR}$ is essential for fixed-parameter tractable algorithms (Linz and Semple 2011; Whidden et al. 2013) and facilitates proofs for $\mathcal{N}\mathcal{P}$-hardness of computing $\textrm{SPR}$ distances, as it is related to the formulation of this problem as an agreement forest problem.

Here we show that $\textrm{RSPR}$ and $\textrm{HSPR}$ space have neither the weak nor the strong cluster property. This observation is important as it suggests that the proving technique for $\mathcal{N}\mathcal{P}$-hardness for rooted (unranked) $\textrm{SPR}$, which uses maximum agreement forests, cannot be used for ranked trees.

#### Theorem 7

$\textrm{HSPR}$ space and $\textrm{RSPR}$ space do not have the weak cluster property.

#### Proof

We prove this theorem by considering trees *T* and *R* that share a cluster but have no shortest path between them that preserves this cluster. Let *T* and *R* be the following two trees (see Fig. 6):

$$\begin{aligned} T = [\{l_1, l_2\}, \{l_1, l_2, l_3\}, \{l_4, l_5\}] \\ R = [\{l_4, l_5\}, \{l_2, l_3\}, \{l_1, l_4, l_5\}] \end{aligned}$$

On any path between *T* and *R* preserving the shared cluster $\{l_4,l_5\}$, the rank of the node inducing this cluster needs to decrease from 3 in *T* to 1 in *R*. Note that originating from *T*, no $\textrm{HSPR}$ move can decrease the rank of this cluster, the only possible moves preserving $\{l_4, l_5\}$ are rank moves. There is hence no shortest path between *T* and *R* in $\textrm{HSPR}$ that preserves $\{l_4,l_5\}$, i.e. $\textrm{HSPR}$ does not have the weak cluster property.

In $\textrm{RSPR}$, two rank moves are necessary to decrease the rank of the node inducing $\{l_4, l_5\}$ to 1, resulting in a tree $R' = [\{l_4, l_5\}, \{l_1, l_2\}, \{l_1, l_2, l_3\}]$. Since $R'$ is not identical to *R*, further $\textrm{RSPR}$ moves are needed to receive *R*, resulting in a path of length greater than two.

There is however a path from *T* and *R* with only two $\textrm{HSPR}$ moves, where first the leaf $l_1$ is pruned and moved to the edge $(\textrm{parent}(l_5), l_5)$ and then $l_5$ is pruned and moved to the edge $(\textrm{parent}(l_4), l_4)$ (see Fig. 6). Since this path is shorter than any path in $\textrm{RSPR}$ preserving the cluster $\{l_4,l_5\}$, $\textrm{RSPR}$ space does not have the weak cluster property. $\square$

## Shortest Paths

One of the main hindrances of using tree rearrangement based distance measures is that most of them are $\mathcal{N}\mathcal{P}$-hard to compute. This applies to rooted and unrooted $\textrm{SPR}$ distance (Bordewich and Semple 2005; Hickey et al. 2008) as well as $\textrm{NNI}$ distance (DasGupta et al. 2000). A recent modification of $\textrm{NNI}$ tree rearrangements for ranked trees has however shown to be computationally tractable (Collienne and Gavryushkin 2021). Whether the same is true for ranked $\textrm{SPR}$ is a natural question, and we approach it by investigating the structure of shortest paths in $\textrm{RSPR}$ and $\textrm{HSPR}$ treespace. Though the complexity of computing distances in $\textrm{HSPR}$ and $\textrm{RSPR}$ remains unknown, our results in this section are a first step towards understanding shortest paths in these treespaces. In particular, we investigate the relationship of shortest paths in $\textrm{HSPR}$ and $\textrm{RSPR}$, which hints at the complexity of the shortest path problem in the two treespaces being identical. Gaining insights into the structure of shortest paths might furthermore be useful for developing algorithms for computing or approximating ranked $\textrm{SPR}$ distances.

### $\textrm{HSPR}$ Shortest Paths

Shortest paths between trees in $\textrm{HSPR}$ are generally not unique, and we show here that they can be arranged so that the rank at which subtrees are cut does not decrease on a shortest path (Theorem 8). We then use this result to study scenarios at which clusters are preserved along shortest paths: First, we show that if the cherry at rank one is identical in two trees, it is preserved on every shortest path (Theorem 9). Then, by further generalising this result, we show in Corollary 4 that for two trees with identical clusters up to a certain rank, all shortest paths between them preserve these clusters. These results provide insights into the shape of shortest paths in $\textrm{HSPR}$, and in future research we hope to leverage these results to prove the complexity of computing distances in this treespace.

To prove that there is a shortest path in $\textrm{HSPR}$ on which ranks of $\textrm{HSPR}$ moves do not decrease, we need the following lemma.

#### Lemma 2

Let $p = [T, T', R]$ be a path in $\textrm{HSPR}$ such that *T* and $T'$ are connected by an $\textrm{HSPR}$ move at rank *k* and $T'$ and *R* are connected by an $\textrm{HSPR}$ move at rank *i* with $k > i$. Then there is a path $p' = [T, T'', R]$ where an $\textrm{HSPR}$ move at rank *i* connects *T* and $T''$ and an $\textrm{HSPR}$ move at rank *k* connects $T''$ and *R*.

#### Proof

As described in Theorem 2, we can describe the $\textrm{HSPR}$ move between trees *T* and $T'$ by the change in the set of edges *E*(*T*), compared to $E(T')$:

$$\begin{aligned} E(T') = E(T) \setminus \{(k^+, k), (k, k^-), (l^+, l^-)\} \cup \{(k^+, k^-), (l^+, k), (k, l^-)\} \end{aligned}$$

where $(k^+, k), (k, k^-), (l^+, l^-) \in E(T)$ and $(l^+, l^-)$ covers rank *k*. Similarly, we assume that $E(T')$ and *E*(*R*) are related in the following way:

$$\begin{aligned} E(R) = E(T') \setminus \{(i^+, i), (i, i^-), (j^+, j^-)\} \cup \{(i^+, i^-), (j^+, i), (i, j^-)\} \end{aligned}$$

where $(i^+, i), (i, i^-), (j^+, j^-) \in E(T')$ and $(j^+, j^-)$ covers rank *i*. In total, we get

$$\begin{aligned} E(R)&= E(T) \cup \big ( \{(k^+, k^-), (l^+, k), (k, l^-)\} \cup \{(i^+, i^-), (j^+, i), (i, j^-)\} \big ) \\&= \setminus \big ( \{(k^+, k), (k, k^-), (l^+, l^-)\} \cup \{(i^+, i), (i, i^-), (j^+, j^-)\} \big ). \end{aligned}$$

Note that there might be edges that are added to $T'$ and then deleted in *R*. Therefore, when summarising multiple $\textrm{HSPR}$ moves as set operations, we assume that we consider multisets, even though the edge set of a tree does not contain an edge multiple times. Let

$$\begin{aligned} E_1&= \{(k^+, k), (k, k^-), (l^+, l^-)\}, E_2 = \{(k^+, k^-), (l^+, k), (k, l^-)\} \\ E_3&= \{(i^+, i), (i, i^-), (j^+, j^-)\},\text { and } E_4 = \{(i^+, i^-), (j^+, i), (i, j^-)\}. \end{aligned}$$

Then $E_3 \cap E_1 = \emptyset$, as otherwise the $\textrm{HSPR}$ move between $T'$ and *R* would not be possible.

If $E_2 \cap E_3 = \emptyset$, then $E_3 \subset E(T)$, and we can create a tree $T''$ with $E(T'') = E(T) \setminus E_3 \cup E_4$, i.e. *T* and $T''$ are connected by an $\textrm{HSPR}$ move at rank *i*. With $E_2 \cap E_3 = \emptyset$ we get $E_2 \in E(T'')$ and we can perform an $\textrm{HSPR}$ move at rank *k* on $T''$ that results in $E(R^*) = E(T'') \setminus E_2 \cup E_1$.

Hence, $E(R^*) = E(T) \cup (E_3 \cup E_1) \setminus (E_4 \cup E_2) = E(R)$. Therefore, $R^*$ and *R* are identical and the path $p' = [T, T'', R]$ contains an $\textrm{HSPR}$ move at rank *i* followed by an $\textrm{HSPR}$ move at rank *k*.

We now distinguish different cases in which $E_2 \cap E_3 \ne \emptyset$. With ${i^-, j^- \prec i \prec k \prec k^+}$, it is $(i, i^-) \notin E_2$ and $(l^+, k) \notin E_3$. Therefore, $E_2 \cap E_3 \ne \emptyset$ if and only if $\{(i^+, i), (j^+, j^-)\} \cap \{(k^+, k^-), (k, l^-)\} \ne \emptyset$. We distinguish six different cases, based on this intersection. In every case, we construct an alternative path $p' = [T, T'', R]$, where *T* and $T''$ are connected by an $\textrm{HSPR}$ move at rank *i* and $T''$ and *R* are connected by an $\textrm{HSPR}$ move at rank *k*. To show that the moves that we describe by edge set changes are valid $\textrm{HSPR}$ moves, we need to show that the edge set changes we provide fulfil the criteria listed in Theorem 2. By our assumption of the moves on *p*, we know that $E_1 \subset E(T)$ and if $e \in E_3 \setminus E_2$, then $e \in E(T)$.

1. $(i^+, i) = (k^+, k^-)$ and $(j^+, j^-) \ne (k, l^-)$: We perform an $\textrm{HSPR}$ move on *T* to receive the tree $T''$ with
  $$\begin{aligned} E(T'') = E(T) \setminus \{(k,i), (i, i^-), (j^+, j^-)\} \cup \{(k, i^-), (j^+, i), (i, j^-)\}. \end{aligned}$$
  This is a valid move because:
  $$(k,i) \in E(T)$$, because $$(k, i) = (k, k^-)$$ and $$(k, k^-) \in E_1$$.
  $$(i, i^-) \in E(T)$$, because $$(i, i^-) \in E_3$$ and $$(i, i^-) \notin E_2$$.
  $$(j^+, j^-) \in E(T)$$, because $$(j^+, j^-) \in E_3$$, and $$(j^+, j^-) \notin E_2$$
  $(j^+, j^-)$ covers rank *i* by the definition of the moves on *p*.
  We can then receive a tree $R^*$ by an $\textrm{HSPR}$ move on $T''$ with
  $$\begin{aligned} E(R^*) = E(T'') \setminus \{(k^+, k), (k, i^-), (l^+, l^-)\} \cup \{(k^+, i^-), (l^+, k), (k, l^-)\}. \end{aligned}$$
  This is a valid move because:
  $(k^+,k) \in E(T'')$, because $(k^+, k) \in E_1$ and $(k^+, k)$ has not been removed between *T* and $T''$.
  $(k, i^-) \in E(T'')$, as it has been added between *T* and $T''$.
  $(l^+, l^-) \in E(T'')$, because $(l^+, l^-) \in E_1$, and $(l^+, l^-)$ has not been removed between *T* and $T''$.
  $(l^+, l^-)$ covers rank *k* by the definition of the moves on *p*.
  With $i^+ = k^+$ and $i = k^-$ it follows $E(R^*) = E(R)$, and hence $R^* \simeq R$.
2. $(i^+, i) = (k^+, k^-)$ and $(j^+, j^-) = (k, l^-)$: Let $T''$ be resulting from *T* by an $\textrm{HSPR}$ move at rank *i* that changes *E*(*T*) as follows:
  $$\begin{aligned} E(T'') = E(T) \setminus \{(k, i), (i, i^-), (l^+, l^-)\} \cup \{(k, i^-), (l^+, i), (i, l^-)\}. \end{aligned}$$
  This is a valid move because:
  $$(k,i) \in E(T)$$, because $$(k, i) = (k, k^-)$$ and $$(k, k^-) \in E_1$$.
  $$(i, i^-) \in E(T)$$, because $$(i, i^-) = (k^-, i^-) \in E_3$$ and $$(k^-, i^-) \notin E_2$$.
  $$(l^+, l^-) \in E(T)$$, because $$(l^+, l^-) \in E_1$$.
  $(j^+, j^-)$ covers rank *i* by the definition of the moves on *p*.
  We can then receive a tree $R^*$ by an $\textrm{HSPR}$ move on $T''$ with
  $$\begin{aligned} E(R^*) = E(T'') \setminus \{(k^+, k), (k, i^-), (l^+, i)\} \cup \{(k^+, i^-), (l^+, k), (k, i)\}. \end{aligned}$$
  This is a valid move because:
  $(k^+,k) \in E(T'')$, because $(k^+, k) \in E_1$ and $(k^+, k)$ has not been removed between *T* and $T''$.
  $(k, i^-) \in E(T'')$, as it has been added between *T* and $T''$.
  $(l^+, i) \in E(T'')$, as it has been added between *T* and $T''$.
  $(l^+, i)$ covers rank *k* by the definition of the moves on *p*.
  With $i^+ = k^+$, $i = k^-$, $j^+ = k$, and $j^- = l^-$ it follows $E(R^*) = E(R)$ and hence $R^* \simeq R$.
3. $(i^+, i) = (k, l^-)$ and $(j^+, j^-) \ne (k^+, k^-)$: We can perform an $\textrm{HSPR}$ move at rank *i* on *T* that gives us the following tree $T''$:
  $$\begin{aligned} E(T'') = E(T) \setminus \{(l^+, i), (i, i^-), (j^+, j^-)\} \cup \{(l^+, i^-), (j^+, i), (i, j^-)\}. \end{aligned}$$
  This is a valid move because:
  $$(l^+,i) \in E(T)$$, because $$(l^+,i) = (l^+, l^-) \in E_1$$.
  $$(i, i^-) \in E(T)$$, because $$(i, i^-) \in E_3$$ and $$(i, i^-) \notin E_2$$.
  $$(j^+, j^-) \in E(T)$$, because $$(j^+, j^-) \in E_3$$ and $$(j^+, j^-) \notin E_2$$.
  $(j^+, j^-)$ covers rank *i* by the definition of the moves on *p*.
  We can then perform an $\textrm{HSPR}$ move at rank *k* on $T''$ to get a tree $R^*$ with
  $$\begin{aligned} E(R^*) = E(T'') \setminus \{(k^+, k), (k, k^-), (l^+, i^-)\} \cup \{(k^+, k^-), (l^+, k), (k, i^-)\}. \end{aligned}$$
  This is a valid move because:
  $(k^+,k) \in E(T'')$, because $(k^+, k) \in E_1$ and $(k^+, k)$ has not been removed between *T* and $T''$.
  $(k, k^-) \in E(T'')$, because $(k^+, k) \in E_1$ and $(k^+, k)$ has not been removed between *T* and $T''$.
  $(l^+, i^-)\in E(T'')$, as it has been added between *T* and $T''$.
  $(j^+, j^-)$ covers rank *k* by the definition of the moves on *p*.
  By $i^+ = k$ and $i = l^-$ it then follows $E(R^*) = E(R)$ and hence $R^* \simeq R$.
4. $(i^+, i) = (k, l^-)$ and $(j^+, j^-) = (k^+, k^-)$: We can perform an $\textrm{HSPR}$ move at rank *i* on *T* to get a tree $T''$ with
  $$\begin{aligned} E(T'') = E(T) \setminus \{(l^+, i), (i, i^-), (k, j^-)\} \cup \{(l^+, i^-), (k, i), (i, j^-)\}. \end{aligned}$$
  This is a valid move because:
  $$(l^+,i) \in E(T)$$, because $$(l^+,i) = (l^+, l^-) \in E_1$$.
  $$(i, i^-) \in E(T)$$, because $$(i, i^-) \in E_3$$ and $$(i, i^-) \notin E_2$$.
  $$(k, j^-) \in E(T)$$, because $$(k, j^-) = (k, k^-) \in E_1$$.
  $(k, j^-)$ covers rank *i* by the definition of the moves on *p*.
  An $\textrm{HSPR}$ move at rank *i* can transform $T''$ to a tree $R^*$ with
  $$\begin{aligned} E(R^*) = E(T'') \setminus \{(k^+, k), (k, i), (l^+, i^-)\} \cup \{(k^+, i), (l^+, k), (k, i^-)\}. \end{aligned}$$
  This is a valid move because:
  $(k^+,k) \in E(T'')$, because $(k^+, k) \in E_1$ and $(k^+, k)$ has not been removed between *T* and $T''$.
  $(k, i) \in E(T'')$, as it has been added between *T* and $T''$.
  $(l^+, i^-) \in E(T'')$, as it has been added between *T* and $T''$.
  $(l^+, i^-)$ covers rank *k* by the definition of the moves on *p*.
  By $i^+ = k$, $i = l^-$, $j^+ = k^+$, and $j^- = k^-$ it follows $E(R^*) = E(R)$ and therefore $R^* \simeq R$.
5. $(j^+, j^-) = (k^+, k^-)$ and $(i^+, i) \ne (k, l^-)$: Performing an $\textrm{HSPR}$ move at rank *i* on *T* can give us a tree $T''$ with
  $$\begin{aligned} E(T'') = E(T) \setminus \{(i^+, i), (i, i^-), (k, j^-)\} \cup \{(i^+, i^-), (k, i), (i, j^-)\}. \end{aligned}$$
  This is a valid move because:
  $$(i^+,i) \in E(T)$$, because $$(i^+,i) \in E_3$$ and $$(i^+,i) \notin E_2$$.
  $$(i, i^-) \in E(T)$$, because $$(i, i^-) \in E_3$$ and $$(i, i^-) \notin E_2$$.
  $$(k, j^-) \in E(T)$$, because $$(k, j^-) = (k, k^-) \in E_1$$.
  $(k, j^-)$ covers rank *i* by the definition of the moves on *p*.
  An $\textrm{HSPR}$ move at rank *k* can therefore convert $T''$ into a tree $R^*$ with
  $$\begin{aligned} E(R^*) = E(T'') \setminus \{(k^+, k), (k, i), (l^+, l^-)\} \cup \{(k^+, i), (l^+, k), (k, l^-)\}. \end{aligned}$$
  This is a valid move because:
  $(k^+,k) \in E(T'')$, because $(k^+, k) \in E_1$ and $(k^+, k)$ has not been removed between *T* and $T''$.
  $(k, i) \in E(T'')$, as it has been added between *T* and $T''$.
  $(l^+, l^-) \in E(T'')$, because $(l^+, l^-) \in E_1$ and $(l^+, l^-)$ has not been removed from *T* to get $T''$.
  $(l^+, l^-)$ covers rank *k* by the definition of the moves on *p*.
  By $j^+ = k^+$ and $j^- = k^-$ it follows $E(R^*) = E(R)$ and hence $R^* \simeq R$.
6. $(j^+, j^-) = (k, l^-)$ and $(i^+, i) \ne (k^+, k^-)$: We can perform an $\textrm{HSPR}$ move at rank *i* on *T* to get a tree $T''$ with edge set
  $$\begin{aligned} E(T'') = E(T) \setminus \{(i^+, i), (i, i^-), (l^+, l^-)\} \cup \{(i^+, i^-), (l^+, i), (i, l^-)\}. \end{aligned}$$
  This is a valid move because:
  $$(i^+,i) \in E(T)$$, because $$(i^+,i) \in E_3$$ and $$(i^+,i) \notin E_2$$.
  $$(i, i^-) \in E(T)$$, because $$(i, i^-) \in E_3$$ and $$(i, i^-) \notin E_2$$.
  $$(l^+, l^-) \in E(T)$$, because $$(l^+, l^-) \in E_1$$.
  $(l^+, l^-)$ covers rank *i* because $i \prec k \prec l^+$ and $j^- = l^- \prec i$.
  Then an $\textrm{HSPR}$ move at rank *k* is possible on $T''$ and transforms this tree into $R^*$ with
  $$\begin{aligned} E(R^*) = E(R) \setminus \{(k^+, k), (k, k^-), (l^+, i)\} \cup \{(k^+, k^-), (l^+, k), (k, i)\}. \end{aligned}$$
  This is a valid move because:
  $(k^+,k) \in E(T'')$, because $(k^+, k) \in E_1$ and $(k^+, k)$ has not been removed between *T* and $T''$.
  $(k, k^-) \in E(T'')$, as $(k, k^-) \in E_1$ and $(k, k^-)$ has not been removed between *T* and $T''$.
  $(l^+, i) \in E(T'')$, because it has been added by the move between *T* and $T''$.
  $(l^+, i)$ covers rank *k* by the definition of the moves on *p*.
  By $j^+ =k$ and $j^- = l^-$, it follows $E(R*) = E(R)$, hence $R^* \simeq R$.

$$\square $$

#### Theorem 8

There is always a shortest $\textrm{HSPR}$ path between any two trees *T* and *R* on which the rank of $\textrm{HSPR}$ moves increases monotonically.

#### Proof

Let *p* be a shortest path between trees *T* and *R*. By Lemma 2 we can take any pair of consecutive $\textrm{HSPR}$ moves at ranks *k* and *i* and if $k > i$, we can replace them by two $\textrm{HSPR}$ moves so that first a rank *i* and then a rank *k* $\textrm{HSPR}$ move is performed. By replacing all such pairs of $\textrm{HSPR}$ moves iteratively, we receive a path on which the ranks of $\textrm{HSPR}$ moves increase monotonically. $\square$

To show that all shortest paths preserve a shared cherry at rank one (Theorem 9), we need the following two lemmas, which describe $\textrm{HSPR}$ moves at a fixed rank *k* along a shortest path. These lemmas are interesting on their own as they are informative about the local geometry of the $\textrm{HSPR}$ treespace. Recall that $(T)_i$ is the node of rank *i* in *T* whereas $T|_i$ is the subtree induced by that node.

#### Lemma 3

Let *T* and *R* be trees containing subtrees $T|_1, T|_2, \dots , T|_d$ with $T|_j \not \simeq T|_m$ for all $j \ne m$, so that $(T)_{i_j}$ and $(R)_{i_j}$ induce the subtree $T|_j$ for $j = 1, \dots , d$ in *T* and *R*, respectively. Let $(i_j^+, i_j)$ be the edges between $(T)_{i_j}$ and its parent in *T* for all $j=1,\dots , d$. If

(i) $i_j \prec \min \limits _{k = 1, \dots , d}(i^+_k)$ for all $j =1, \dots , d$ and
(ii) $$\begin{aligned} E(R) = E(T) \cup \{(i_1^+, i_d)\} \bigcup _{j = 1, \dots , d-1}\{(i_{j+1}^+, i_j)\} \setminus \Big ( \bigcup _{j = 1, \dots , d}\{(i_j^+, i_j)\} \Big ), \end{aligned}$$

Then $$d_{\textrm{HSPR}}(T,R) \le d$$.

Informally, the difference between *T* and *R* is the positioning of the subtrees $T|_1, T|_2, \dots , T|_d$, which we can interpret as a permutation of these subtrees. By changing every edge $(i_j^+, i_j)$ of *T* to $(i_{j+1}^+, i_j)$ in *R*, every subtree $T|_j$ in *R* is attached where $T|_{j+1}$ is in *T* for all $j = 1, \dots , d-1$, and with $(i_1^+, i_1)$ being replaced by $(i_1^+, i_d)$, $T|_d$ in *R* is attached where $T|_1$ is in *T*. We could describe this permutation of the subtrees by $(T|_1, T|_2, \dots , T|_d)$, using the cycle notation for permutations.

#### Proof

We assume without loss of generality that $i_1^+ = \min \limits _{j = 1, \dots , d} i_j^+$. If otherwise $i_m^+ = \min \limits _{j = 1, \dots , d} i_j^+$ for $m \ne 1$, we rename the subtrees $T|_{((j+d-m+1) \bmod d) + 1}$ to $T|_j$ for all $j = 1, \dots , d$. By the assumptions of the lemma, all edges $(i_j, i^+_j)$ with $j \ne i$ in *T* cover rank $k := i_1^+$. Let $i_1^{++} = \textrm{parent}_T(k)$ and let $i_0$ be the sibling of $i_1$ in *T*, i.e. $\textrm{parent}_T(i_0) = \textrm{parent}_T(i_1) = k$.

We now create a path $p = [T_0 \simeq T, T_1, \dots , T_d \simeq R]$ of length *d*. We describe the moves on *p* iteratively for every pair $T_{j-1}, T_j$ by using the edge set notation. For every move we make, we prove that it is a valid move by showing that the conditions of Theorem 2 are fulfilled. Note that we can infer from $T|_j \not \simeq T|_m$ that $i_j \ne i_m$ for all $j \ne m$.

We define the first move on *p* by the following change of the edge set of *T*:

$$\begin{aligned} E(T_1) = E(T)&\setminus \{(i_1^{++}, k), (k, i_0), (i_2^+, i_2)\} \\&\cup \{(i_1^{++}, i_0), (i_2^+, k), (k, i_2)\}. \end{aligned}$$

This is a valid move, because:

- $(i_1^{++}, k) \in E(T)$ by definition of $i_1^{++}$ and *k*.
- $(k, i_0) \in E(T)$ by definition of *k* and $i_0$.
- $(i_2^+, i_2) \in E(T)$ by the assumption of the lemma.
- $(i_2^+, i_2)$ covers rank *k* by the assumptions of the lemma.

The next moves on *p* between $T_{j-1}$ and $T_j$ for $j = 2, \dots , d-1$ are described by the following changes in edge sets:

$$\begin{aligned} E(T_j) = E(T_{j-1})&\setminus \{(i_j^+, k), (k, i_{j-1}), (i_{j+1}^+, i_{j+1})\} \\&\cup \{(i_j^+, i_{j-1}), (i_{j+1}^+, k), (k, i_{j+1})\}. \end{aligned}$$

These are valid moves, because:

- $(i_j^+, k) \in E(T_{j-1})$, because this edge is added to $E(T_{j-1})$ by the previous move between $T_{j-2}$ and $T_{j-1}$.
- $(k, i_{j-1}) \in E(T_{j-1})$, because this edge is added in the move from $T_{j-3}$ to $T_{j-2}$, and it is not changed when moving from $T_{j-2}$ to $T_{j-1}$.
- $(i_{j+1}^+, i_{j+1}) \in E(T_{j-1})$, because $(i_{j+1}^+, i_{j+1}) \in E(T)$ by the assumptions of the lemma, and with $i_j \ne i_m$ for all $j \ne m$ and $i_{j+1}^+ \ne k$ for all $j \ne 1$, this edge is not changed on any previous move on *p*.
- $(i_{j+1}^+,i_{j+1})$ covers rank *k* by the assumption of the lemma.

The last step, transforming $T_{d-1}$ to $T_d$, we apply an $\textrm{HSPR}$ move to $T_{d-1}$ that changes the edge set as follows:

$$\begin{aligned} E(T_d) = E(T_{d-1})&\setminus \{(i_d^+, k), (k, i_{d-1}), (i_1^{++}, i_0)\} \\&\cup \{(i_d^+, i_{d-1}), (i_1^{++}, k), (k, i_0)\}. \end{aligned}$$

This is a valid move, because:

- $(i_d^+, k) \in E(T_{d-1})$, because this edge is added to $E(T_{d-1})$ by the previous move between $T_{d-2}$ and $T_{d-1}$.
- $(k, i_{d-1}) \in E(T_{d-1})$, because this edge is added in the move from $T_{d-3}$ to $T_{d-2}$, and it is not changed when moving from $T_{d-2}$ to $T_{d-1}$.
- $(i_1^{++}, i_0) \in E(T_{d-1})$, because it is added to $E(T_1)$ in the first move on *p* and with $i_j \ne i_m$ for all $j \ne m$ and $i_1^{++} \ne k$, we can infer that it is not removed from the edge set of any tree on any previous move on *p*.
- $i_0 \prec k \prec i_1^{++}$ by definition of *k*.

We can summarise all changes to the edge sets along *p* using multisets, which can be simplified using $k = i_1^+$:

$$\begin{aligned} E(T_d) = E(T)&\cup \{(i_1^{++}, i_0), (i_2^+, k), (k, i_2)\} \\&\cup \bigcup _{j = 2, \dots , d-1} \{(i_j^+, i_{j-1}), (i_{j+1}^+, k), (k, i_{j+1})\} \\&\cup \{(i_d^+, i_{d-1}), (i_1^{++}, k), (k, i_0)\} \\&\setminus \{(i_1^{++}, k), (k, i_0), (i_2^+, i_2)\} \\&\setminus \Big ( \bigcup _{j=2, \dots , d-1} \{(i_j^+, k), (k, i_{j-1}), (i_{j+1}^+, i_{j+1})\} \Big ) \\&\setminus \{(i_d^+, k), (k, i_{d-1}), (i_1^{++}, i_0)\} \\ = E(T)&\cup \{(i_1^+, i_d)\} \cup \bigcup _{j = 2, \dots , d} \{(i_j^+, i_{j-1})\} \\&\setminus \bigcup _{j=1, \dots , d} \{(i_j^+, i_j)\}. \end{aligned}$$

It follows $E(T_d) = E(R)$ and therefore, $T_d \simeq R$. Hence, *p* is a path from *T* to *R* of length *d*, proving $d_{\textrm{HSPR}}(T,R) \le d$. $\square$

**Fig. 7:**
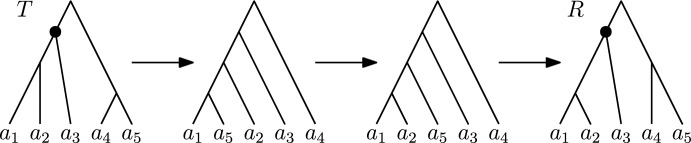
Shortest $\textrm{HSPR}$ path between trees *T* and *R*. The subtree consisting of only the leaf $a_5$ is moved by the first and the last $\textrm{HSPR}$ move. The path displayed here does not preserve the shared cluster $\{a_1, a_2, a_3\}$ at rank three. The nodes inducing this cluster are highlighted in *T* and *R*. On this path only subtrees consisting of single leaves move

Another interesting property of shortest paths is that of Lemma 4: No subtree can move twice by $\textrm{HSPR}$ moves at the same rank. It is however possible for one subtree to move twice at different ranks on a shortest path. For example in Fig. 7, the subtree only consisting of the leaf $a_5$ is moved by the first $\textrm{HSPR}$ move at rank one and by the last $\textrm{HSPR}$ move at rank two.

#### Lemma 4

Let *T* and *R* be trees connected by a shortest path *p* containing only $\textrm{HSPR}$ moves at rank *k*, moving subtrees $T|_{i_0}, T|_{i_1}, \dots , T|_{i_{d-1}}$ in this order, i.e. *p* has length *d*.

Then every $T|_{i_j}$ is subtree of both *T* and *R* and $T|_{i_j} \not \simeq T|_{i_l}$ for all $j \ne l \in \{0, 1, \dots , d-1\}$.

#### Proof

Let *p* be a shortest path only containing $\textrm{HSPR}$ moves at rank *k* as described in the lemma. Every subtree $T|_{i_j}$ with $j \in \{0, 1, \dots , d-1\}$ is induced by a node $i_j$ with rank less than *k* in *T*. Therefore, no edges inside any of these subtrees can change on *p*, which implies that $T|_{i_0}, \dots , T|_{i_{d-1}}$ are subtrees in all trees on *p*, including *T* and *R*.

To show $T|_{i_j} \not \simeq T|_{i_l}$ for all $j \ne l$, we assume to the contrary that a subtree $T' \in \{T|_{i_0}, \dots , T|_{i_{d-1}}\}$ moves twice on a *p*. To simplify notation, we assume without loss of generality that it is $T' \simeq T|_{i_0} \simeq T|_{i_{d-1}}$ and that no other subtree moves twice on *p*. Note that it could be $d-1 = 1$, in which case $T'$ moves twice in two consecutive moves.

Let $i_j^+ = \textrm{parent}_T(i_j)$ in *T*. Since the first move on *p* moves the subtree $T|_{i_0}$ and all moves on *p* are $\textrm{HSPR}$ moves at rank *k*, it is $i_0 = k$. Furthermore, the subtree $T|_{i_j}$ needs to have node *k* as its parent before it gets pruned from a tree on *p*, as all moves are $\textrm{HSPR}$ moves at rank *k*. This implied that $T|_{i_j}$ gets reattached on the edge $(i_{j+1}^+, i_{j+1})$ for every $j = 0, \dots , d-2$ to ensure that the next move can prune the subtree $T|_{i_{j+1}}$ at rank *k*. Let $i_0$ be the sibling of $i_1$ in *T*, i.e. $\textrm{parent}_T(i_0) = \textrm{parent}_T(i_1) = k$ and let $i_1^{++} = \textrm{parent}_T(i_1^+)$.

Assuming $p = [T_0 \simeq T, T_1, \dots , T_d \simeq R]$, the $\textrm{HSPR}$ moves along *p* can formally be described by the following changes in edge sets (see Theorem 3):

$$\begin{aligned} E(T_1) = E(T)&\setminus \{(i_1^{++}, k), (k, i_0), (i_2^+, i_2)\} \\&\cup \{(i_1^{++}, i_0), (i_2^+, k), (k, i_2)\} \end{aligned}$$

and for $$j = 2, \dots , d-2$$:

$$\begin{aligned} E(T_j) = E(T_{j-1})&\setminus \{(i_j^+, k), (k, i_{j-1}), (i_{j+1}^+, i_{j+1})\} \\&\cup \{(i_j^+, i_{j-1}), (i_{j+1}^+, k), (k, i_{j+1})\} \end{aligned}$$

By our assumptions on *p*, $T|_{i_j} \not \simeq T|_{i_m}$ for all $j \ne m$ in $\{1, \dots , d-1\}$, we know that $(i_{j+1}^+, i_{j+1}) \in E(T_{j-1})$ for all $j = 1, \dots , d-1$. We can use similar arguments to those used in the proof of Lemma 3 to show that the changes of edge sets presented here describe valid $\textrm{HSPR}$ moves up to $T_{d-2}$.

For the last move on *p*, moving the subtree $T|_{i_d} \simeq T|_{i_0} \simeq T'$ by an $\textrm{HSPR}$ move at rank *k*, the subtree $T|_{i_{d-1}}$ needs to become sibling of $T|_{i_d}$ by an $\textrm{HSPR}$ move at rank *k* between $T_{d-2}$ and $T_{d-1}$. The edge connecting $i_1$, which is the root of $T|_{i_d} \simeq T|_{i_0}$, and its parent is $(i_2^+, i_1)$, which is created by the move between $T_1$ and $T_2$ and with $i_2^+ \ne k$ and $i_j \ne i_k$ for all $j \ne k$ in $\{1, \dots , d-1\}$, this edge is not changed again on *p* until reaching $T_{d-2}$. The move between $T_{d-2}$ and $T_{d-1}$ can therefore be described by the following change in the edge set of $T_{d-2}$:

$$\begin{aligned} E(T_{d-1}) = E(T_{d-2})&\setminus \{(i_{d-1}^+, k), (k, i_{d-2}), (i_2^+, i_1)\} \\&\cup \{(i_{d-1}^+, i_{d-2}), (i_2^+, k), (k, i_1)\} \end{aligned}$$

Let $(i_{d+1}^+, i_{d+1})$ be the edge on which $T' \simeq T_s^d$ gets reattached by the last move on *p*. We can then write this last move as

$$\begin{aligned} E(T_d \simeq R) = E(T_{d-1})&\setminus \{(i_2^+, k), (k, i_{d-1}), (i_{d+1}^+, i_{d+1})\} \\&\cup \{(i_2^+, i_{d-1}), (i_{d+1}^+, k), (k, i_{d+1})\}. \end{aligned}$$

It is again not hard to see with Theorem 2 that this describes a valid $\textrm{HSPR}$ move.

Using multisets, we can describe the difference between *E*(*R*) and *E*(*T*) as follows:

$$\begin{aligned} E(R) = E(T)&\cup \{(i_1^{++}, i_0), (i_2^+, k), (k, i_2)\} \\&\cup \bigcup _{j = 2, \dots , d-2} \{(i_j^+, i_{j-1}), (i_{j+1}^+, k), (k, i_{j+1})\} \\&\cup \{(i_{d-1}^+, i_{d-2}), (i_2^+, k), (k, i_1)\} \\&\cup \{(i_2^+, i_{d-1}), (i_{d+1}^+, k), (k, i_{d+1})\} \\&\setminus \{(i_1^{++}, k), (k, i_0), (i_2^+, i_2)\} \\&\setminus \Big ( \bigcup _{j=2, \dots , d-2} \{(i_j^+, k), (k, i_{j-1}), (i_{j+1}^+, i_{j+1})\} \Big ) \\&\setminus \{(i_{d-1}^+, k), (k, i_{d-2}), (i_2^+, i_1)\} \\&\setminus \{(i_2^+, k), (k, i_{d-1}), (i_{d+1}^+, i_{d+1})\} \\ = E(T)&\cup \{(i_1^{++}, i_0),(i_2^+, i_{d-1}), (i_{d+1}^+, k), (k, i_{d+1})\} \\&\cup \bigcup _{j = 3, \dots , d-1} \{(i_j^+, i_{j-1})\} \\&\setminus \{(i_1^{++}, k), (k, i_0),(i_{d+1}^+, i_{d+1})\} \\&\setminus \Big ( \bigcup _{j=2, \dots , d-1} \{(i_j^+, i_j)\} \Big ). \end{aligned}$$

Based on this, we can define an alternative path from *T* to *R*. Therefore, we define a neighbour ${\hat{T}}$ of *T* by

$$\begin{aligned} E({\hat{T}}) = E(T)&\setminus \{(i_1^{++}, k), (k, i_0), (i_{d+1}^+, i_{d+1})\} \\&\cup \{(i_1^{++}, i_0), (i_{d+1}^+, k), (k, i_{d+1})\}. \end{aligned}$$

This describes a valid $\textrm{HSPR}$ move on *T*, because:

- $(i_1^{++}, k) \in E(T)$ by the definition of $i_1^{++}$ and *k*.
- $(k, i_0) \in E(T)$ by the definition of *k* and $i_0$.
- $(i_{d+1}^+, i_{d+1}^-) \in E(T)$, because $(i_{d+1}^+, i_{d+1}^-) \in E(T_{d-1})$ by the definition of moves on *p* and this edge is not deleted from the edge set on any move on *p* before $T_{d-1}$ is reached.
- $(i_{d+1}^+, i_{d+1}^-)$ covers rank *k* by the assumption that all $\textrm{HSPR}$ move are $\textrm{HSPR}$ moves at rank *k*.

Knowing the difference between *E*(*T*) and *E*(*R*), we can summarise the difference between *E*(*R*) and $E({\hat{T}})$ to:

$$\begin{aligned} E(R) = E({\hat{T}})&\cup \{(i_2^+, i_{d-1})\} \\&\cup \bigcup _{j = 3, \dots , d-1} \{(i_j^+, i_{j-1})\} \\&\setminus \Big ( \bigcup _{j=2, \dots , d-1} \{(i_{j}^+, i_{j})\} \Big ). \end{aligned}$$

${\hat{T}}$ and *R* fulfil the assumptions of Lemma 3, so we get $d_{\textrm{HSPR}}({\hat{T}},R) = d-2$. Therefore, the path from *T* to *R* via ${\hat{T}}$ has length $1 + d-2 = d-1$ and is shorter than *p*, contradicting our assumption that *p* is a shortest path. Hence, no subtree is moved twice on a shortest with only $\textrm{HSPR}$ moves at fixed rank *k*. $\square$

We are now ready to show that a shared cherry at rank one is preserved on every shortest path.

#### Theorem 9

Let *T* and *R* be trees and $x,y \in \{l_1, \dots , l_n\}$ so that both *T* and *R* have $\{x,y\}$ as cherry at rank one. Then every tree on every shortest path between *T* and *R* has $\{x, y\}$ as cherry at rank one.

#### Proof

For this proof we assume to the contrary that *T* and *R* are connected by a shortest path *p* containing a tree whose cherry at rank one is not $\{x,y\}$. We furthermore assume that *T* and *R* have minimum distance among all tree pairs connected by such a shortest path. It then follows that *p* contains $\textrm{HSPR}$ moves at rank one only: Otherwise, we could change the order of ranks of $\textrm{HSPR}$ moves along the path to have all rank one $\textrm{HSPR}$ moves first (Lemma 2). Since the cherry at rank one cannot change by $\textrm{HSPR}$ moves at rank greater than one, *T* and *R* would then not be a minimum distance counterexample. Therefore, all $\textrm{HSPR}$ moves on *p* are at rank one and hence move subtrees that consist of one leaf only.

**Fig. 8:**
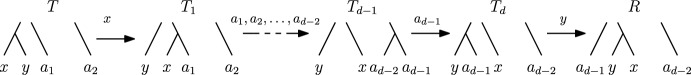
Trees *T* and *R* with cherry $\{x, y\}$ at rank one and all moves on a path *p* from *T* to *R* that does not preserve the cherry as described in the proof of Theorem 9. The labels of the arrows indicate the leaves that are pruned and reattached by the corresponding $\textrm{HSPR}$ moves

By the assumption on *T* and *R*, either *x* or *y* is pruned by the first move on *p*. We assume that *x* is moved first, otherwise we swap notations for *x* and *y*. Since the last move on *p* reconstructs the cherry $\{x, y\}$ at rank one, and the subtree containing only the leaf *x* can only move once at rank one (Lemma 4), the last leaf that moves on *p* is *y*. Let $a_0 = x, a_1, a_2, \dots , a_{d-1}, a_d = y$ be the sequence of leaves moved on *p*. By Lemma 4, all these leaves are distinct i.e. $a_i \ne a_j$ for all $i \ne j$. Note that $d-1 \ge 1$, as after the first move on *p* the parent of *y* has rank greater than one, but the last move on *p* moves *y* by an $\textrm{HSPR}$ move at rank one, for which the parent of *y* needs to have rank one. Moving $a_{d-1} \ne x$ to become sibling of *y* in the second to last move is hence necessary to get to *R*, so $d-1 \ge 1$. The path *p* as described above is depicted in Fig. 8.

Let $a_i^+ = \textrm{parent}_T(a_i)$ for all $i = 1, \dots , d$ and $p_{xy} = \textrm{parent}_T(1)$. Note that this implies $a_i^+ \ne 1$ for all *i*. Assuming $p = [T_0 \simeq T, T_1, \dots , T_d, T_{d+1} \simeq R]$, we can then describe the moves on *p* similar to how it has been done in the proof of Lemma 4:

$$\begin{aligned} E(T_1) = E(T_0)&\setminus \{(p_{xy}, 1), (1, y), (a_1^+, a_1)\} \\&\cup \{(p_{xy}, y), (a_1^+, 1), (1, a_1)\}. \end{aligned}$$

This describes a valid move, since:

- $(p_{xy}, 1) \in E(T_0)$ by the definition of $p_{xy}$.
- $(1, y) \in E(T_0)$, because *y* is in the cherry of rank one.
- $(a_1^+, a_1) \in E(T_0)$ by the definition of $a_1^+$.
- $(a_1^+, a_1)$ covers rank 1, because $a_1$ is a leaf and $a_1^+ \ne 1$.

$$\begin{aligned} E(T_j) = E(T_{j-1})&\setminus \{(a_{j-1}^+, 1), (1, a_{j-2}), (a_j^+, a_j)\} \\&\cup \{(a_{j-1}^+, a_{j-2}), (a_j^+, 1), (1, a_j)\} \end{aligned}$$

for $$j = 2, \dots , d-1$$.

This describes a valid move, because:

- $(a_{j-1}^+, 1) \in E(T_{j-1})$, because it has been added to this set by the previous move between $T_{j-2}$ and $T_{j-1}$.
- $(1, a_{j-2}) \in E(T_{j-1})$, because it has been added to $E(T_{j-2})$ by the move between $T_{j-3}$ and $T_{j-2}$ and with $a_i \ne a_j$ for all $i \ne j$, this edge has not been removed from $E(T_{j-2})$ to get $E(T_{j-1})$.
- $(a_j^+, a_j) \in E(T_{j-1})$, because $(a_j^+, a_j) \in E(T)$ and with $a_i \ne a_j$ for all $i \ne j$, this edge is not removed from the edge set in any previous move on *p*.
- $(a_j^+, a_j)$ covers rank 1, because $a_j$ is a leaf and $a_j^+ \ne 1$.

$$\begin{aligned} E(T_d) = E(T_{d-1})&\setminus \{(a_{d-1}^+, 1), (1, a_{d-2}), (p_{xy}, y)\} \\&\cup \{(a_{d-1}^+, a_{d-2}), (p_{xy}, 1), (1, y)\} \end{aligned}$$

This describes a valid move, because:

- $(a_{d-1}^+, 1) \in E(T_{d-2})$, because it has been added to this set by the previous move between $T_{d-3}$ and $T_{d-2}$.
- $(1, a_{d-2}) \in E(T_{d-2})$, because it has been added to $E(T_{d-3})$ by the move between $T_{d-2}$ and $T_{d-2}$.
- $(p_{xy}, y) \in E(T_{d-2})$, because it has been added to $E(T_1)$ by the first move on *p* and with $y \ne a_i, 1$ for all *i*, this edge is not removed from the edge set in any previous move on *p*.
- $(p_{xy},y)$ covers rank 1, because $(p_{xy}, 1)$ and (1, *y*) are edges in *T*.

$$\begin{aligned} E(R) = E(T_d)&\setminus \{(p_{xy}, 1), (1, a_{d-1}), (a_1^+, x)\} \\&\cup \{(p_{xy}, a_{d-1}), (a_1^+, 1), (1, x)\} \end{aligned}$$

This describes a valid move, because:

- $(p_{xy}, 1) \in E(T_d)$, because it has been added to this set by the previous move between $T_{d-1}$ and $T_d$.
- $(1, a_{d-1}) \in E(T_d)$, because it has been added to $E(T_{d-2})$ by the move between $T_{d-2}$ and $T_{d-1}$ and with $a_i \ne a_j$ for all $i \ne j$, this edge has not been removed from $E(T_{d-1})$ to get $E(T_d)$.
- $(a_1^+, x) \in E(T_{j-1})$, because it has been added to $E(T_2)$ by the second move on *p* ($x = a_0$) and with $x \ne a_i, 1$ for all *i*, this edge is not removed from the edge set in any previous move on *p*.
- $(a_1^+, x)$ covers rank 1, because *x* is a leaf and $1 \prec a_i^+$ for all *i*.

Since some edges that are added along *p* are later deleted, we can describe the changes between *E*(*T*) and *E*(*R*) by using multisets:

$$\begin{aligned} E(R) := E(T)&\cup \{(p_{xy}, y), (a_1^+, 1), (1, a_1)\} \\&\cup \bigcup _{j=2, \dots , d-1} \{(a_{j-1}^+, a_{j-2}), (a_j^+, 1), (1, a_j)\} \\&\cup \{(a_{d-1}^+, a_{d-2}), (p_{xy}, 1), (1, y)\} \\&\cup \{(p_{xy}^+, a_{d-1}), (a_1^+, 1), (1, x)\} \\&\setminus \{(p_{xy}, 1), (1, y), (a_1^+, a_1)\} \\&\setminus \Big ( \bigcup _{j=2, \dots , d-1} \{(a_{j-1}^+, 1), (1, a_{j-2}), (a_j^+, a_j)\} \Big ) \\&\setminus \{(a_{d-1}^+, 1), (1, a_{d-2}), (p_{xy}, y)\} \\&\setminus \{(p_{xy}, 1), (1, a_{d-1}), (a_1^+, x)\}, \\ \end{aligned}$$

which we can summarise to:

$$\begin{aligned} E(R) =&E(T) \cup \Big (\{(a_1^+, 1), (p_{xy}, a_{d-1})\} \bigcup _{j=1, \dots , d-2} \{(a_{j+1}^+, a_j)\} \Big ) \\&\setminus \Big ({(p_{xy}, 1)} \bigcup _{j=1, \dots , d-1} \{(a_j^+, a_j)\} \Big ). \\ \end{aligned}$$

*T* and *R* fulfil the requirements of the trees of Lemma 3, where $i_j^+ = a_j^+$ and $i_j = a_j$ for all $j = 1, \dots , d-1$, $i_d^+ = p_{xy}$, and $i_d = 1$. Applying this lemma gives us $d_{\textrm{HSPR}}(T,R) \le d$.

Therefore, *p* is not shortest paths, contradicting our assumption that there is a shortest path from *T* to *R* that does not preserve the shared cherry. $\square$

Theorem 9 implies that the distance of two trees with identical cherry at rank one does not change when one of the leaves in this cherry is deleted (Corollary 3). We will see in Theorem 11 that we can generally not assume that the distance stays the same or decreases when deleting a leaf from two trees.

#### Corollary 3

Let *T* and *R* be trees on *n* leaves sharing their cherry $\{c_1, c_2\}$ at rank one and let $T'$ and $R'$ result from deleting $c_2$ from *T* and *R*, respectively, suppressing the internal node of rank one and subtracting one from the rank of all remaining internal nodes so that *T* and *R* are trees on $n-1$ leaves. Then $d_{\textrm{HSPR}}(T',R') = d_{\textrm{HSPR}}(T,R)$.

#### Proof

By Theorem 9 all shortest $\textrm{HSPR}$ paths from *T* to *R* preserve the cherry $\{c_1,c_2\}$. Therefore, deleting $c_2$ from every tree on a shortest *T*-*R*-path, suppressing the resulting degree-two nodes, and subtracting one from the ranks of all remaining nodes, gives a path between $T'$ and $R'$, i.e. $d_{\textrm{HSPR}}(T',R') \le d_{\textrm{HSPR}}(T,R)$. If there was a path between $T'$ and $R'$ that was shorter than $d_{\textrm{HSPR}}(T,R)$, then adding a leaf $c_2$ as sibling of $c_1$ with parent of rank one and adding one to the ranks of all other internal nodes in every tree on *p* results in a path between *T* and *R* of length less than $d_{\textrm{HSPR}}(T,R)$, which is a contradiction. Hence, $d_{\textrm{HSPR}}(T,R) = d_{\textrm{HSPR}}(T',R')$. $\square$

Another observation that follows from Theorem 9 is that if the lowest part of two trees, i.e. all clusters up to a certain rank, is identical, then no shortest path in $\textrm{HSPR}$ changes this part of the tree.

#### Corollary 4

Let *T* and *R* be trees so that for some $i \in \{2, \dots , n\}$ the cluster induced by $(T)_j$ is identical to the cluster induced by $(R)_j$ for all $j < i$. Then every node $(T')_j$ in every tree $T'$ on every shortest path between *T* and *R* induces the same cluster as $(T)_j$ and $(R)_j$ for all $j < i$.

#### Proof

To prove this corollary, we assume to the contrary that there is a node with rank less than *i* that induces the same cluster in *T* and *R*, but this cluster is not present on a shortest path *p* between *T* and *R*. Let *j* be the rank of such a node so that there is no other node with this property in *T* and *R* with rank less than *j*. Then all clusters induced by nodes of rank less than *j* are present in all trees on *p*. We can iteratively apply Corollary 3 to the trees *T* and *R* where in the first iteration deleting a cherry leaf and updating the rest of the tree as described in the lemma results in trees $T_1$ and $R_1$ with $d_{\textrm{HSPR}}(T, R) = d_{\textrm{HSPR}}(T_1, R_1)$. This can be repeated until trees $T_{j-1}$, $R_{j-1}$ are received with $d_{\textrm{HSPR}}(T_{j-1}, R_{j-1}) = d_{\textrm{HSPR}}(T,R)$, where $T_{j-1}$ and $R_{j-1}$ are trees on $n-(j-1)$ leaves. As all clusters induced by nodes of rank less than *j* are present in all trees on *p*, the leaves that are deleted from *T* and *R* to receive $T_{j-1}$ and $R_{j-1}$, respectively, can be deleted in the same order in all trees on *p* in the same way, and we receive a path $p'$ from $T_{j-1}$ to $R_{j-1}$ with $|p'| = |p|$. Since the cluster induced by the nodes of rank *j* is the same in *T* and *R*, $T_{j-1}$ and $R_{j-1}$ have the same cherry at rank one. Because *p* does not preserve the cluster at rank *j*, there must be a tree on $p'$ not containing the cherry of rank one in $T_{j-1}$ and $R_{j-1}$, which implies by Corollary 3 that $p'$ is not a shortest path, i.e. $d_{\textrm{HSPR}}(T_{j-1}, R_{j-1}) < |p'| = |p|$. This however is a contradiction to $d_{\textrm{HSPR}}(T_{j-1}, R_{j-1}) = d_{\textrm{HSPR}}(T,R) = |p|$. Therefore, there cannot be a shortest path *p* between *T* and *R* that does not preserve the cluster of rank $j<i$ that is present in *T* and *R*, which concludes the proof of this corollary. $\square$

It is in general not true that a cluster *C* that is induced by nodes of the same rank *r* in two trees *T* and *R* is present in all trees on all shortest paths. A counterexample to this can be found in Fig. 7 where the shared cluster $\{a_1, a_2, a_3\}$ at rank three is not present in every tree on any shortest path from *T* to *R*, which we found out through exhaustive search using our implementation (Collienne 2023).

### $\textrm{RSPR}$ Shortest Paths

The $\textrm{RSPR}$ space can be interpreted as an extension of $\textrm{HSPR}$ in which rank moves are added to provide shortcuts between some trees. Here, we investigate how the addition of rank moves changes shortest paths. Our analysis of these paths will provide insights into the relationship of the complexity of computing distances in $\textrm{HSPR}$ and $\textrm{RSPR}$. We show in Theorem 10 that we can change the order of moves on paths in $\textrm{RSPR}$, while not changing their length, so that all rank moves are grouped at the beginning followed by a sequence of only $\textrm{HSPR}$ moves. This indicates that shortest paths in $\textrm{HSPR}$ and $\textrm{RSPR}$ can be very similar, sometimes identical (e.g. in the case of caterpillar trees, Corollary 6), and suggests that the complexity of computing shortest paths (and distances) might be the same in both treespaces. We first need the following lemma:

#### Lemma 5

Let *T* and *R* trees and $p = [T, T', R]$ a path with an $\textrm{HSPR}$ move between *T* and $T'$ and a rank move between $T'$ and *R*. Then there is a path $p' = [T, T'', R]$ consisting of either two $\textrm{HSPR}$ moves or a rank move followed by an $\textrm{HSPR}$ move.

Whether the path $p'$ in Lemma 5 contains two $\textrm{HSPR}$ moves or a rank move followed by an $\textrm{HSPR}$ move depends on the specific moves on *p*, as we will see in the proof of this lemma. For this proof we use the cluster representation of trees and describe $\textrm{HSPR}$ moves by the corresponding changes in cluster representation as described in Theorem 3.

#### Proof

**Fig. 9:**
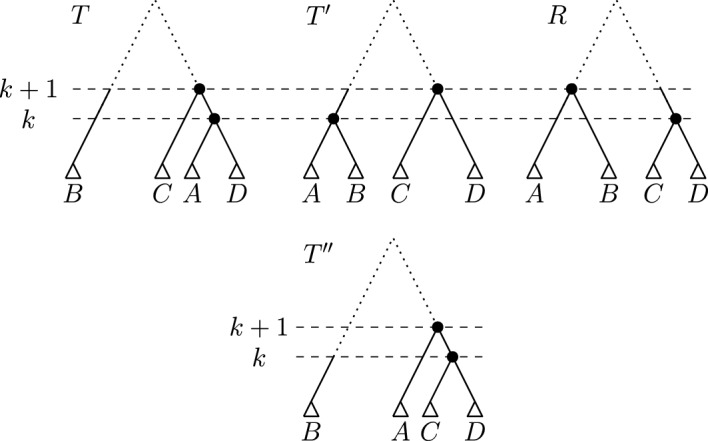
Trees *T*, $T'$, and *R* on *p*, and alternative path $p'$ from *T* to *R* via $T''$ at the bottom if *T* and $T'$ are connected by an $\textrm{HSPR}$ move at rank *k* and the nodes of rank *k* and $k+1$ in *T* are connected by an edge, as explained in case 2.1. The dotted parts of the trees might contain further nodes and leaves

Let *k* and $k+1$ for some $k \in \{1, \dots , n-3\}$ be the ranks of the nodes of the rank move between $T'$ and *R*. Let $T' = [C_1, \dots , C_{k-1}, A \cup B, C \cup D, C_{k+2}, \dots , C_{n-1}]$ be the cluster representation of $T'$, which is illustrated in the top middle of Fig. 9. Since $T'$ and *R* are connected by a rank move of nodes *k* and $k+1$, the cluster representation of *R* is $R = [C_1, \dots , C_{k-1}, C \cup D, A \cup B, C_{k+2}, \dots , C_{n-1}]$ (see top right of Fig. 9).

In the following, we distinguish different $\textrm{HSPR}$ moves possible between *T* and $T'$ and show how to replace $T'$ by a tree $T''$ to get a path $p' = [T, T'', R]$ with either two $\textrm{HSPR}$ moves or a rank move followed by an $\textrm{HSPR}$ move.

**Fig. 10:**
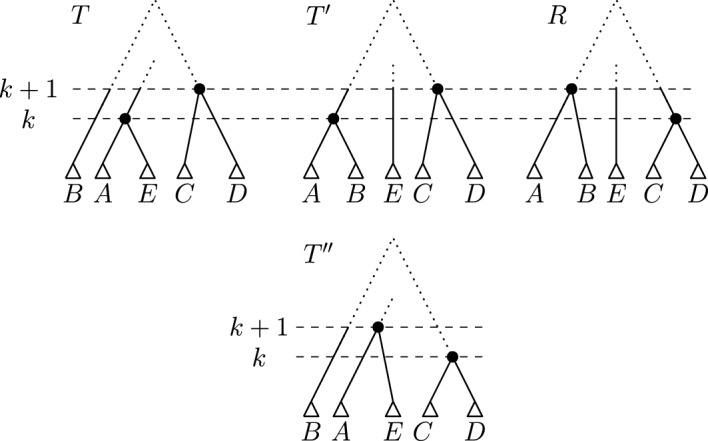
Trees *T*, $T'$, and *R* on *p* at the top, and alternative path $p'$ from *T* to *R* via $T''$ at the bottom if *T* and $T'$ are connected by an $\textrm{HSPR}$ move at rank *k* and the nodes of rank *k* and $k+1$ in *T* are not connected by an edge. The dotted parts of the trees might contain further nodes and leaves

1. The $\textrm{HSPR}$ move between *T* and $T'$ is neither a rank *k* nor a rank $k+1$ $\textrm{HSPR}$ move. An $\textrm{HSPR}$ move at rank greater than *k* does not change the clusters induced by nodes *k* and $k+1$, so we can in this case first perform a rank move of nodes *k* and $k+1$ on *T* to get a tree $T''$. Since all clusters of rank greater than $k+1$ are identical in *T* and $T''$ we can perform an $\textrm{HSPR}$ move on $T''$ that changes the clusters of this tree in exactly the same way as they change between *T* and $T'$. This $\textrm{HSPR}$ move on $T''$ then results in *R*, giving us a path $p' = [T, T'', R]$ with the desired properties. If the $\textrm{HSPR}$ move between *T* and $T'$ is an $\textrm{HSPR}$ move at rank $r < k$, it might change the clusters induced by the nodes of rank *k* and $k+1$. It does however not matter in which order these two clusters appear in the tree, they would change in the exact same way if they were swapped. Therefore, we can first swap the ranks of the nodes with rank *k* and $k+1$ in *T*, resulting in a tree $T''$, and then perform the same $\textrm{HSPR}$ move on $T''$ as the one between *T* and $T'$. This results in a path $p'' = [T, T'', R]$ with a rank move between *T* and $T''$ and an $\textrm{HSPR}$ move between $T''$ and *R*.
2. *T* and $T'$ are connected by an $\textrm{HSPR}$ move at rank *k*. Without loss of generality we can assume that $T|_A$ is moved between *T* and $T'$, otherwise we swap notations for *A* and *B*. We now further distinguish whether there is an edge connecting the nodes of rank *k* and $k+1$ in *T*.
  2.1. Let there be an edge connecting the nodes with ranks *k* and $k+1$ in *T*. Note that by our assumptions on $T'$, $\textrm{parent}_{T'}(T|_C) = \textrm{parent}_{T'}(T|_D) = k+1$ and $\textrm{parent}_{T'}(T|_A) = k$. Therefore, an $\textrm{HSPR}$ move at rank *k* on $T'$ that moves the subtree $T|_A$ and creates a tree *T* containing an edge $(k+1,k)$ must move $T|_A$ to become sibling of either $T|_C$ or $T|_D$. We assume without loss of generality that $T|_A$ and $T|_D$ are siblings in *T*, as depicted in Fig. 9, otherwise we change notation for *C* and *D*. Then the cluster representation of *T* is:
    $$\begin{aligned} T = [C_1, \dots , C_{k-1}, A \cup D, A \cup C \cup D, C'_{k+2}, \dots , C'_{n-2}], \end{aligned}$$
    where for all $m = k+1, \dots , n-2$:
    $$\begin{aligned} C'_m = {\left\{ \begin{array}{ll} C_m \setminus A &{} \quad \text { if } A \subset C_m \text { and } D \not \subset C_m \\ C_m \cup A &{} \quad \text { if } D \subset C_m \\ C_m &{} \quad \text { otherwise}. \end{array}\right. } \end{aligned}$$
    To get a path $p'$ with the desired properties, we perform an $\textrm{HSPR}$ move at rank *k* on *T* that moves $T|_D$ to become sibling of $T|_C$, resulting in a tree $T''$ in which the parent of $T''|_A$ has rank $k+1$. This tree $T''$ has cluster representation:
    $$\begin{aligned} T'' = [C_1, \dots , C_{k-1}, C \cup D, A \cup C \cup D, C''_{k+2}, \dots , C''_{n-1}] \end{aligned}$$
    where for all $m = k+1, \dots , n-2$:
    $$\begin{aligned} C''_m = {\left\{ \begin{array}{ll} C'_m \setminus D &{} \quad \text { if } D \subset C'_m \text { and } C \not \subset C'_m \\ C'_m \cup D &{} \quad \text { if } C \subset C'_m \\ C'_m &{} \quad \text { otherwise}. \end{array}\right. } \end{aligned}$$
    Remember that every cluster is the union of two clusters at lower rank and/or leaves of a tree. Since the node $(T)_{k+1}$ induced the cluster $A \cup C \cup D$, it is $A \subset C'_m$ if and only if $(C \cup D) \subset C'_m$ for all $m \ge k+1$. Therefore, all clusters induced by nodes with rank greater than $k+1$ are the same in *T* and $T''$: $C''_m = C'_m$ for all $m = k+2, \dots , n-1$. We can now perform an $\textrm{HSPR}$ move at rank $k+1$ on $T''$ that moves the subtree $T''|_A$ to become sibling of $T''|_B$, which results in a tree ${\hat{R}}$ with cluster representation:
    $$\begin{aligned} {\hat{R}} = [C_1, \dots , C_{k-1}, C \cup D, A \cup B, {\hat{C}}_{k+2}, \dots , {\hat{C}}_{n-2}] \end{aligned}$$
    where for all $m=k+1, \dots , n-2$:
    $$\begin{aligned} {\hat{C}}_m = {\left\{ \begin{array}{ll} C'_m \setminus A &{} \quad \text { if } A \subset C'_m \text { and } B \not \subset C'_m \\ C'_m \cup A &{} \quad \text { if } B \subset C'_m \\ C'_m &{} \quad \text { otherwise}. \end{array}\right. } \end{aligned}$$
    Using the fact that $B \subset C_m$ if and only if $B \subset C'_m$, we can summarise the change of a cluster at rank $m > k+1$ between *T* and ${\hat{R}}$ as follows:
    $$\begin{aligned} {\hat{C}}_m = {\left\{ \begin{array}{ll} C_m \setminus A &{} \quad \text { if } A \subset C_m \text { and } B \not \subset C_m \text { and } D \not \subset C_m \\ C_m \cup A &{} \quad \text { if } B \subset C_m \text { and } D \subset C_m \\ C_m &{} \quad \text { otherwise}. \end{array}\right. } \end{aligned}$$
    Since the cluster at rank *k* in $T'$ is $A \cup B$, it is $A \subset C_m$ if and only if $B \subset C_m$ for all $m > k$. Therefore, ${\hat{C}}_m = C_m$ for all $m > k+1$. It follows that the cluster representation of ${\hat{R}}$ and *R* coincides, which gives us ${\hat{R}} \simeq R$, so $p'$ is a path from *T* to *R* with two $\textrm{HSPR}$ moves (see Fig. 9).
  2.2. If there is no edge between the nodes with rank *k* and $k+1$ in *T*, then there is a cluster *E* in *T* so that $T|_A$ is sibling of $T|_E$ in *T* with $E \ne A, B, C, D$ (see Fig. 10). Then the cluster representation of *T* is:
    $$\begin{aligned} T = [C_1, \dots , C_{k-1}, A \cup E, C \cup D, C'_{k+2}, \dots , C'_{n-1}] \end{aligned}$$
    where for all $m = k+1, \dots , n-2$:
    $$\begin{aligned} C'_m = {\left\{ \begin{array}{ll} C_m \setminus A &{} \quad \text { if } A \subset C_m \text { and } E \not \subset C_m \\ C_m \cup A &{} \quad \text { if } E \subset C_m \\ C_m &{} \quad \text { otherwise}. \end{array}\right. } \end{aligned}$$
    To create an alternative path $p'$, we first perform a rank move swapping the ranks of the nodes *k* and $k+1$ in *T* to receive a tree $T''$ with cluster representation:
    $$\begin{aligned} T'' = [C_1, \dots , C_{k-1}, C \cup D, A \cup E, C'_{k+2}, \dots , C'_{n-1}]. \end{aligned}$$
    As second move on $p'$ we perform an $\textrm{HSPR}$ move at rank $k+1$, moving the subtree $T''|_A$ to become sibling of $T''|_B$, giving us the following tree ${\hat{R}}$:
    $$\begin{aligned} {\hat{R}} = [C_1, \dots , C_{k-1}, C \cup D, A \cup B, {\hat{C}}_{k+2}, \dots , {\hat{C}}_{n-1}], \end{aligned}$$
    where clusters ${\hat{C}}_m$ with $m \ge k+2$ are defined as follows:
    $$\begin{aligned} {\hat{C}}_m = {\left\{ \begin{array}{ll} C'_m \setminus A &{} \quad \text { if } A \subset C'_m \text { and } B \not \subset C'_m \\ C'_m \cup A &{} \quad \text { if } B \subset C'_m \\ C'_m &{} \quad \text { otherwise}. \end{array}\right. } \end{aligned}$$

**Fig. 11:**
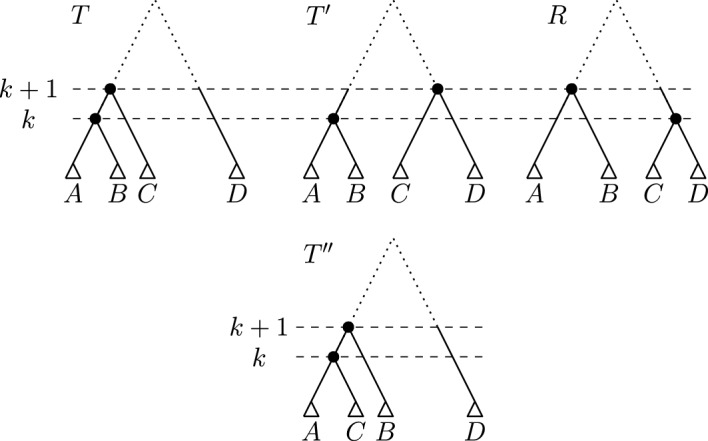
Trees *T*, $T'$, and *R* on *p*, and alternative path $p'$ from *T* to *R* via $T''$ at the bottom if *T* and $T'$ are connected by an $\textrm{HSPR}$ move at rank $k+1$ and the nodes of rank *k* and $k+1$ in *T* are connected by an edge, The dotted parts of the trees might contain further nodes and leaves
    To show that ${\hat{C}}_m = C_m$ for all $m = k+2, \dots , n-1$, we distinguish whether $A \subset C_m$ or $A \not \subset C_m$. Note that $A \subset C_m$ if and only if $B \subset C_m$ and also $B \subset C_m$ if and only if $B \subset C'_m$. If $A \subset C_m$ and $E \not \subset C_m$, then $C'_m = C_m \setminus A$ and since $B \subset C'_m$, ${\hat{C}}_m = C'_m \cup A$, resulting in ${\hat{C}}_m = C_m$. If on the other hand $A \subset C_m$ and $E \subset C_m$, then $C'_m = C_m \cup A$ and with $B \subset C'_m$, ${\hat{C}}_m = C'_m \cup A$, resulting in ${\hat{C}}_m = C_m$. If $A \not \subset C_m$ and $E \subset C_m$, then $C'_m = C_m \cup A$ and since $B \not \subset C'_m$, ${\hat{C}}_m = C'_m \setminus A$, resulting in ${\hat{C}}_m = C_m$. If on the other hand $A \not \subset C_m$ and $E \not \subset C_m$, then $C'_m = C_m$ and since $B \not \subset C'_m$, ${\hat{C}}_m = C_m$, resulting in ${\hat{C}}_m = C_m$. So for every $m = k+2, \dots , n-1$, it is ${\hat{C}}_m = C'_m$, which implies ${\hat{R}} \simeq R$, so $p'$ is a path from *T* to *R* with first a rank move and then an $\textrm{HSPR}$ move.
3. *T* and $T'$ are connected by an $\textrm{HSPR}$ move at rank $k+1$. As $T|_C$ and $T|_D$ are the subtrees that are children of node $k+1$ in $T'$, we can assume without loss of generality that $T|_C$ is moved between *T* and $T'$, otherwise we swap notation for *C* and *D*. We again further distinguish whether the node of rank $k+1$ is parent of the node of rank *k* in *T* or not.
  3.1. Let there be an edge connecting nodes $k+1$ and *k* in *T*. Remember that by our assumptions on $T'$, $\textrm{parent}_{T'}(T|_A) = \textrm{parent}_{T'}(T|_B) = k$. Therefore, an $\textrm{HSPR}$ move at rank $k+1$ on $T'$ that moves the subtree $T|_C$ and creates a tree *T* containing an edge $(k+1,k)$ must move $T|_C$ to become sibling of $T|_{A \cup B}$ (see Fig. 11). Then *T* has the following cluster representation:
    $$\begin{aligned} T = [C_1, \dots , C_{k-1}, A \cup B, A \cup B \cup C, C'_{k+2}, \dots C'_{n-1}], \end{aligned}$$
    where
    $$\begin{aligned} C'_m = {\left\{ \begin{array}{ll} C_m \setminus C &{} \quad \text { if } C \subset C_m \text { and } (A \cup B) \not \subset C_m \\ C_m \cup C &{} \quad \text { if } (A \cup B) \subset C_m \\ C_m &{} \quad \text { otherwise} \end{array}\right. } \end{aligned}$$
    for all $m = k+2, \dots , n-1$. A path $p'$ can now be constructed by first performing an $\textrm{HSPR}$ move at rank *k* on *T* moving $T|_A$ to become sibling of $T|_C$ in the resulting tree $T''$:
    $$\begin{aligned} T'' = [C_1, \dots , C_{k-1}, A \cup C, A \cup B \cup C, C''_{k+2}, \dots , C''_{n-1}] \end{aligned}$$
    with
    $$\begin{aligned} C''_m = {\left\{ \begin{array}{ll} C'_m \setminus A &{} \quad \text { if } A \subset C'_m \text { and } C \not \subset C'_m \\ C'_m \cup A &{} \quad \text { if } C \subset C'_m \\ C'_m &{} \quad \text { otherwise} \end{array}\right. } \end{aligned}$$
    for all $m = k+2, \dots , n-1$. Because the node of rank $k+1$ in *T* induced the cluster $A \cup B \cup C$, $A \subset C'_m$ if and only if $C \subset C'_m$ for all $m = k+2, \dots , n-1$. Therefore, $C''_m = C'_m$ for all $m = k+2, \dots , n-1$. We then perform an $\textrm{HSPR}$ move at rank *k* on $T''$ that moves the subtree $T''|_C$ to become sibling of $T''|_D$, resulting in the following tree ${\hat{R}}$:
    $$\begin{aligned} {\hat{R}} = [C_1, \dots , C_{k-1}, C \cup D, A \cup B, {\hat{C}}_{k+2}, \dots , {\hat{C}}_{n-1}] \end{aligned}$$
    with
    $$\begin{aligned} {\hat{C}}_m = {\left\{ \begin{array}{ll} C'_m \setminus C &{} \quad \text { if } C \subset C'_m \text { and } D \not \subset C'_m \\ C'_m \cup C &{} \quad \text { if } D \subset C'_m \\ C'_m &{} \quad \text { otherwise} \end{array}\right. } \end{aligned}$$
    for all $m = k+2, \dots , n-1$. To show ${\hat{R}} \simeq R$, we use that $C \subset C_m$ if and only if $D \subset C_m$, and $A \subset C_m$ if and only if $B \subset C_m$ for all $m = k+2, \dots , n-1$, because the clusters induced by nodes of rank *k* and $k+1$ in $T'$ are $A \cup B$ and $C \cup D$, respectively. Furthermore, we distinguish whether $(A \cup B) \subset C_m$, $(C \cup D) \subset C_m$, and $(A \cup B \cup C \cup D) \cap C_m = \emptyset$ If $(A \cup B) \subset C_m$ for some $m = k+2, \dots , n-1$, then $C'_m = C_m \cup C$. Then (i) ${\hat{C}}_m = C'_m \setminus C$ if $D \not \subset C'_m$ or (ii) ${\hat{C}}_m = C'_m \cup C$ if $D \subset C'_m$. Note that if $D \subset C'_m$, it must be $D \subset C_m$, as *D* is not removed from any clusters between trees $T'$ and *T*. And since $D \subset C_m$ if and only if $C \subset C_m$, we can see that ${\hat{C}}_m = C_m$ for both cases (i) and (ii). If $(C \cup D) \subset C_m$ for some $m = k+2, \dots , n-1$, then (i) $C'_m = C_m \setminus C$ if $(A \cup B) \not \subset C_m$ or (ii) $C'_m = C_m \cup C$ if $(A \cup B) \subset C_m$. Again, it is $D \subset C'_m$ if and only if $D \subset C_m$. Therefore, it must be ${\hat{C}}_m = C'_m \cup C$ in both cases (i) and (ii). With $C \subset C_m$, it follows ${\hat{C}}_m = C_m$. If $(A \cup B \cup C \cup D) \cap C_m = \emptyset$, then $C'm = C_m$ and ${\hat{C}}_m = C'_m$, hence ${\hat{C}}_m = C_m$. We can conclude that ${\hat{R}} \simeq R$, so $p'$ is a path from *T* to *R* consisting of two $\textrm{HSPR}$ moves.
  3.2. There is no edge between the nodes of rank *k* and $k+1$ in *T*. Let *E* be a cluster in *T* so that the subtree $T|_E$ is sibling of $T|_C$ in *T* with $E \ne A,B,C,D$, i.e. $T|_C$ is moved to become sibling of $T|_E$ by the $\textrm{HSPR}$ move between $T'$ and *T* (see Fig. 12). The cluster notation for *T* is:
    $$\begin{aligned} T = [C_1, \dots , C_{k-1}, A \cup B, C \cup E, C'_{k+2}, \dots , C'_{n-1}] \end{aligned}$$
    with
    $$\begin{aligned} C'_m = {\left\{ \begin{array}{ll} C_m \setminus C &{} \quad \text { if } C \subset C_m \text { and } E \not \subset C_m \\ C_m \cup C &{} \quad \text { if } E \subset C_m \\ C_m &{} \quad \text { otherwise} \end{array}\right. } \end{aligned}$$
    for all $m = k+2, \dots , n-1$. We construct a path $p'$ by first performing a rank move swapping the nodes *k* and $k+1$ of *T*, resulting in the tree $T''$:
    $$\begin{aligned} T'' = [C_1, \dots , C_{k-1}, C \cup E, A \cup B, C'_{k+2}, \dots , C'_{n-1}] \end{aligned}$$
    We can then preform an $\textrm{HSPR}$ move on $T''$, moving the subtree $T''|_C$ to become sibling of $T''|_D$, which gives us the tree ${\hat{R}}$:
    $$\begin{aligned} {\hat{R}} = [C_1, \dots , C_{k-1}, C \cup D, A \cup B, {\hat{C}}_{k+2}, \dots , {\hat{C}}_{n-1}] \end{aligned}$$
    with
    $$\begin{aligned} {\hat{C}}_m = {\left\{ \begin{array}{ll} C'_m \setminus C &{} \quad \text { if } C \subset C'_m \text { and } D \not \subset C'_m \\ C'_m \cup C &{} \quad \text { if } D \subset C'_m \\ C'_m &{} \quad \text { otherwise} \end{array}\right. } \end{aligned}$$
    for all $m = k+2, \dots , n-1$. To show that ${\hat{C}}_m = C_m$ for all $m = k+2, \dots , n-1$, we distinguish whether $C \subset C_m$ or $C \not \subset C_m$. Note that since $C \cup D$ is the cluster induced by node *k* in *T*, it is $C \subset C_m$ if and only if $D \subset C_m$ and since *D* is not removed from any clusters between $T'$ and *T*, also $D \subset C_m$ if and only if $D \subset C'_m$ for all $m = k+2, \dots , n-1$. If $C \subset C_m$ and $E \not \subset C_m$, then $C'_m = C_m \setminus C$ and since $D \subset C'_m$, ${\hat{C}}_m = C'_m \cup C$, resulting in ${\hat{C}}_m = C_m$. If on the other hand $C \subset C_m$ and $E \subset C_m$, then $C'_m = C_m \cup C$ and with $D \subset C'_m$, ${\hat{C}}_m = C'_m \cup C$, resulting in ${\hat{C}}_m = C_m$. If $C \not \subset C_m$ and $E \subset C_m$, then $C'_m = C_m \cup C$ and since $D \not \subset C'_m$, ${\hat{C}}_m = C'_m \setminus C$, resulting in ${\hat{C}}_m = C_m$. If on the other side $C \not \subset C_m$ and $E \not \subset C_m$, then $C'_m = C_m$ and ${\hat{C}}_m = C'_m$, resulting in ${\hat{C}}_m = C_m$. In any case, it is ${\hat{C}}_m = C_m$ for all $m = k+2, \dots , n-1$ and therefore ${\hat{R}} \simeq R$, which implies that $p'$ is a path from *T* to *R* that consists of a rank move followed by an $\textrm{HSPR}$ move.

In all cases above, we found an alternative path $p'$ to the path *p* so that $p'$ consists of either two $\textrm{HSPR}$ moves or a rank move followed by an $\textrm{HSPR}$ move, which concludes the proof of this lemma. $\square$

**Fig. 12:**
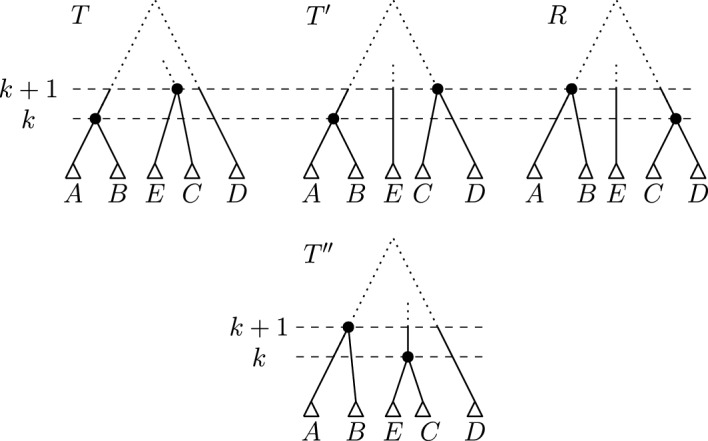
Trees *T*, $T'$, and *R* on *p* at the top, and alternative path $p'$ from *T* to *R* via $T''$ at the bottom if *T* and $T'$ are connected by an $\textrm{HSPR}$ move at rank *k* and the nodes of rank *k* and $k+1$ in *T* are not connected by an edge. The dotted parts of the trees might contain further nodes and leaves

Using Lemma 5, we can now prove that we can change the order of moves on an $\textrm{RSPR}$ path so that in the beginning we have a sequence of rank moves, followed by a sequence of $\textrm{HSPR}$ moves.

#### Theorem 10

In $\textrm{RSPR}$ there is always a shortest path between two trees that has a sequence of rank moves (if there are any) at the beginning followed by only $\textrm{HSPR}$ moves.

#### Proof

To prove the theorem we assume that there is a shortest path $p= [T_0, T_1, \dots , T_d]$ between trees $T_0$ and $T_d$ that has at least one rank move preceded by an $\textrm{HSPR}$ move. Let $T_{i-1}$ and $T_i$ for some $1< i < d -1$ be connected by the first $\textrm{HSPR}$ move on *p* that has a rank move following it, i.e. $T_i$ and $T_{i+1}$ are connected by a rank move. By Lemma 5, we can replace $T_i$ by a tree $T'_i$ so that there is a rank move or $\textrm{HSPR}$ move between $T_{i-1}$ and $T'_i$ and an $\textrm{HSPR}$ move between $T'_i$ and *R*. When iteratively applying this procedure to all rank moves that are preceded by an $\textrm{HSPR}$ move on *p*, we receive a path from *T* to *R* that has all rank moves at the beginning of the path, followed by a sequence of only $\textrm{HSPR}$ moves. $\square$

Theorem 10 implies that any (not necessarily shortest) path between two trees *T* and *R* can be converted into a *T*-*R*-path of the same length that has all rank moves bundled at the beginning of the path, followed by $\textrm{HSPR}$ moves. We can also shuffle moves on an $\textrm{RSPR}$ path in the opposite way so that $\textrm{HSPR}$ moves happen first and rank moves last. Additionally, we can infer the following from Theorem 10.

#### Corollary 5

Let $T_u$ and $R_u$ be rooted (unranked) trees. Then there are ranked trees $T = (T_u, \textrm{rank}_T)$ and $R = (R_u, \textrm{rank}_R)$ so that $d_{\textrm{RSPR}}(T, R) = d_{\textrm{HSPR}}(T, R)$.

#### Proof

Let ${\widehat{\textrm{rank}}}_T$ be an arbitrary rank function of $T_u$, i.e. $T' = (T_u, {\widehat{\textrm{rank}}}_T)$ is a ranked tree, and let $\textrm{rank}_R$ be a rank function on $R_u$ so that $R = (R_u, \textrm{rank}_R)$ is a ranked tree. By Theorem 10, we can find a shortest path *p* from $T'$ to *R* in $\textrm{RSPR}$ that has all rank moves bundled in the beginning of the path. Let *T* be the ranked tree on *p* after this sequence of rank moves, i.e. the remainder of *p* between *T* and *R* consists of $\textrm{HSPR}$ moves only. Since there is a path from $T'$ to *T* consisting of rank moves only, the unranked versions of $T'$ and *T* are identical, i.e. $T = (T_u, \textrm{rank}_T)$ for some rank function $\textrm{rank}_T$ on $T_u$. Furthermore, as *p* is a shortest path in $\textrm{RSPR}$, the part of *p* between *T* and *R* is a shortest path between those trees in $\textrm{RSPR}$. And because this part of *p* consists of $\textrm{HSPR}$ moves only, it must also be a shortest path in $\textrm{HSPR}$. We conclude that there are ranked trees $T = (T_u, \textrm{rank}_T)$ and $R = (R_u, \textrm{rank}_R)$ so that $d_{\textrm{RSPR}}(T, R) = d_{\textrm{HSPR}}(T, R)$. $\square$

#### Corollary 6

If there is a shortest path between trees *T* and *R* in $\textrm{RSPR}$ that contains a caterpillar tree, then there is a shortest path from *T* to *R* that consists of $\textrm{HSPR}$ moves only and $d_\textrm{HSPR}(T,R) = d_\textrm{RSPR}(T,R)$.

#### Proof

Let *T* and *R* be trees connected by a shortest path *p* that contains a caterpillar tree $T_c$. The sequence of trees on *p* between *T* and $T_c$ is a shortest path between these trees, and its reversed order a shortest path from $T_c$ to *T*. Applying Theorem 10 to this path from $T_c$ to *T* gives a shortest path where all rank moves are at the beginning of the path. Since $T_c$ is a caterpillar tree, there cannot be rank moves on $T_c$ which implies that there is a shortest paths from *T* to $T_c$ that consists of $\textrm{HSPR}$ moves only.

By the same argument, the restriction of *p* from $T_c$ to *R* can be transformed into a path of the same length that consists of only $\textrm{HSPR}$ moves.

We can concatenate the shortest path from *T* to $T_c$ with only $\textrm{HSPR}$ moves and the one from $T_c$ and *R* with only $\textrm{HSPR}$ moves and receive a path from *T* to *R* that has the same length as *p*. This path is hence a shortest path and consists of $\textrm{HSPR}$ moves only. $\square$

## Adding leaves

In this section we consider how adding a leaf to two trees can change their distance. Especially when considering data sets of ongoing evolutionary processes, like virus transmissions, it would be ideal to re-use the already inferred tree, and some methods to do this already exist (Gill et al. 2020; Dinh et al. 2018; Fourment et al. 2018; Bouckaert et al. 2022). For analyses where new leaves are added to an already existing tree, it is important to understand how adding a leaf can change a tree. It is also of interest to know how the distance between two trees changes when a new leaf is added. One would naturally assume that the addition of a leaf to two trees increases their distance. For $\textrm{HSPR}$ and $\textrm{RSPR}$, however, we find that adding one leaf can decrease the distance between two trees linearly in *n* (Theorem 11). We will see that this is the case when two trees are similar and the new leaf is added at different heights in the tree. Such leaves with varying positions in a tree are often referred to as “rogue taxa” (Aberer et al. 2013), and our results here show that the existence of these can have a big impact on the distance between two trees under $\textrm{RSPR}$ and $\textrm{HSPR}$.

Before we describe how adding a leaf results in a decrease in distance, we need the following observation.

### Lemma 6

The caterpillar trees

$$\begin{aligned} T&= [\{l_1, l_2\}, \{l_1, l_2, l_3\}, \dots , \{l_1, l_2, l_3, \dots , l_{n-1}\}, \{l_1, l_2, \dots , l_n\}] \text { and} \\ R&= [\{l_2, l_3\}, \{l_2, l_3, l_4\}, \dots , \{l_2, l_3, l_4, \dots , l_n\}, \{l_1, l_2, \dots , l_n\}] \end{aligned}$$

have $\textrm{HSPR}$ and $\textrm{RSPR}$ distance greater than or equal to $\lceil \frac{n-1}{2} \rceil$.

**Fig. 13:**
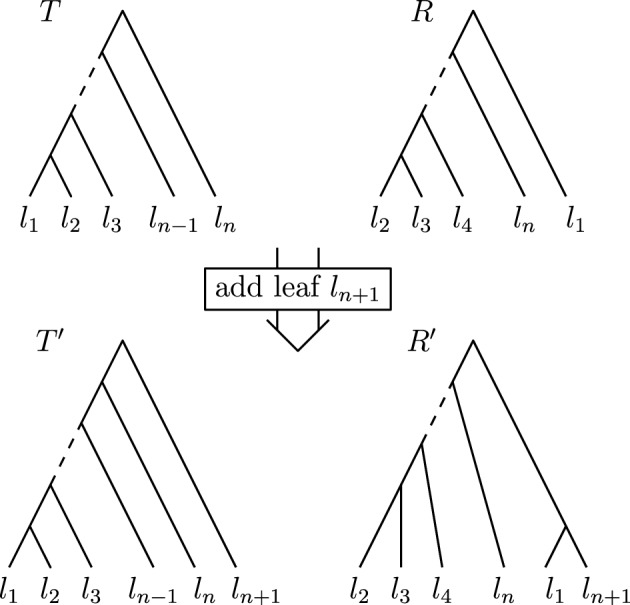
Top: trees *T* and *R* with $\textrm{HSPR}$ distance greater than or equal to $\frac{n-1}{2}$ (Lemma 6). Bottom: Adding leaf $l_{n+1}$ to both *T* and *R* results in trees $T'$ and $R'$ that are connected by one $\textrm{HSPR}$ move that moves $l_1$

The trees *T* and *R* of Lemma 6 are displayed in the top row of Fig. 13. Whether the bound for the distance of *T* and *R* in Lemma 6 is sharp remains an open question.

### Proof

The parents of $n-1$ leaves $l_1, l_3, l_4, \dots , l_n$, i.e. all leaves except $l_2$, have different ranks in *T* and *R*. By Lemma 1 it follows $d_{\textrm{HSPR}}(T,R) \ge \lceil \frac{n-1}{2} \rceil$. And as the $\textrm{HSPR}$ and $\textrm{RSPR}$ distance between caterpillar trees coincides (Corollary 6), $d_{\textrm{RSPR}}(T,R) \ge \lceil \frac{n-1}{2} \rceil$. $\square$

Note that Lemma 6 implies that the diameter of $\textrm{RSPR}$ is greater than or equal to $\lceil \frac{n-1}{2} \rceil$, which is the same boundary we already found for $\textrm{HSPR}$ in Theorem 6. We are now ready to prove the main theorem of this section.

### Theorem 11

Adding a leaf to two trees can decrease their $\textrm{HSPR}$ distance by $\lceil \frac{n-1}{2}\rceil -1$.

### Proof

Let *T* and *R* be the trees of Lemma 6. We add a leaf $l_{n+1}$ to these trees *T* and *R* as follows (see Fig. 13): A new root is added to *T* so that the resulting tree $T'$ has $l_{n+1}$ and the old root of *T* as children. In *R* we attach $l_{n+1}$ as sibling of $l_1$ so that their parent has rank one, and the ranks of all other internal nodes of *R* are increased by one, giving us a tree $R'$ on $n+1$ leaves. The cluster representations of $T'$ and $R'$ are:

$$\begin{aligned} T'&= [\{l_1, l_2\}, \{l_1, l_2, l_3\}, \dots , \{l_1, l_2, \dots , l_n\}, \{l_1, l_2, \dots , l_n, l_{n+1}\}] \text { and} \\ R'&= [\{l_1, l_{n+1}\}, \{l_2, l_3\}, \{l_2, l_3, l_4\}, \dots , \{l_2, l_3, l_4, \dots , l_n\}, \{l_1, l_2, \dots , l_n, l_{n+1}\}]. \end{aligned}$$

Let $C_i$ and $C'_i$ be the cluster induced by the node *i* in $T'$ and $R'$, respectively, for all $i = 1, \dots , n-1$. We can then describe the difference between the cluster representations of $T'$ and $R'$ as follows: $C'_1 = \{l_1, l_{n+1}\}$ and for all $m = 2, \dots , n$:

$$\begin{aligned} C'_m = {\left\{ \begin{array}{ll} C_m \setminus \{l_1\} &{} \quad \text { if } \{l_1\} \subset C_m \text { and } \{l_{n+1}\} \not \subset C_m \\ C_m \cup \{l_{1}\} &{} \quad \text { if } \{l_{n+1}\} \subset C_m\\ C_m &{} \quad \text { otherwise}. \end{array}\right. } \end{aligned}$$

By Theorem 3, $T'$ and $R'$ are connected by an $\textrm{HSPR}$ move.

Therefore, adding one leaf to *T* and *R* changes the distance from $d_{\textrm{HSPR}}(T,R) \ge \lceil \frac{n-1}{2} \rceil$ (Lemma 6) to $d_{\textrm{HSPR}}(T',R') = 1$, which means that their distance decreases by at least $\lceil \frac{n-1}{2} \rceil -1$. $\square$

In Theorem 12 we will see that adding a leaf can only increase the $\textrm{HSPR}$ distance if the unranked versions of the given trees are connected by a rooted (unranked) $\textrm{SPR}$ move.

### Theorem 12

Let $T = (T_u, \textrm{rank}_R)$ and $R = (R_u, \textrm{rank}_R)$ be trees with $\textrm{HSPR}$ distance $d_{\textrm{HSPR}}(T,R) = d > 1$, and let $T'$ and $R'$ be trees resulting from adding a leaf *x* to *T* and *R*, respectively, so that $d_{\textrm{HSPR}}(T',R') = 1$.

If $T_u$ and $R_u$ are the unranked versions of *T* and *R*, respectively, then the rooted (unranked) $\textrm{SPR}$ distance of $T_u$ and $R_u$ is one: $d_{\textrm{SPR}}(T_u,R_u) = 1$.

### Proof

Since $T'$ and $R'$ have $\textrm{HSPR}$ distance one, there is a subtree $T|_i$ present in $T'$ and $R'$ that is moved between them. It follows directly from the definitions of $\textrm{SPR}$ and $\textrm{HSPR}$ moves that if $T'$ and $R'$ are connected by an $\textrm{HSPR}$ move, their unranked versions $T'_u$ and $R'_u$ are connected by an $\textrm{SPR}$ move, too, and the subtree moving between $T'_u$ and $R'_u$ is the unranked version $T|_i^u$ of $T|_i$.

To show $d_\textrm{SPR}(T_u, R_u) = 1$, we compare the distance of $T_u$ and $R_u$ with that of $T'_u$ and $R'_u$ and analyse how removing leaf *x* from $T'_u$ and $R'_u$ to obtain $T_u$ and $R_u$ affects their distance. We know that $T'_u$ and $R'_u$ differ only in the position of subtree $T|_i^u$. We consider following cases, depending on the position of the leaf *x* relative to $T|_i^u$:

(i) If $T|_i^u$ has *x* as its only leaf, then $T_u \simeq R_u$, contradicting the assumptions of the theorem.
(ii) If *x* is not in $T|_i^u$, then $T_u$ and $R_u$ differ only by the position of $T|_i^u$.
(iii) If *x* is not the only leaf in the leaf set of $T|_i^u$, then $T_u$ and $R_u$ differ only by the position of $T|_i^u - x$.

While case (i) is not possible under our assumptions, case (ii) and (iii) both imply that $T_u$ and $R_u$ are connected by one $\textrm{SPR}$ move. Therefore, we conclude that $d_\textrm{SPR}(T_u, R_u) = 1$. $\square$

## Discussion

Many tree inference methods use $\textrm{SPR}$ moves for tree proposals, and extensive research made the $\textrm{SPR}$ treespace usable for analysing tree inference methods and their output. There have however not been any comparable studies for phylogenetic time trees, even though time trees are inferred from sequence data in many applications and software packages like BEAST2 (Bouckaert et al. 2014) for time tree inference are extremely popular. One approach to considering $\textrm{SPR}$ moves for time trees is that by Song (2006), where $\textrm{SPR}$ moves are allowed to re-attach subtrees at the same height or closer to the root than its previous attachments. With this paper, we introduce two ranked $\textrm{SPR}$ treespaces, $\textrm{RSPR}$ and $\textrm{HSPR}$, that are motivated by the variations of $\textrm{SPR}$ moves that are actually used as tree proposals in practice and have some properties in common with rooted (unranked) $\textrm{SPR}$. We show that all of these treespaces are connected, have neighbourhood sizes quadratic in the number of leaves *n*, and have a diameter linear in *n*. These properties have already been proven to be useful for $\textrm{SPR}$, which provides a good range of different trees for tree proposals (Whidden and Matsen 2015). Adding ranks to $\textrm{SPR}$ provides an even more biologically relevant distance measure, as it ensures that the times of nodes in subtrees cannot change by one $\textrm{HSPR}$ move, which particularly makes sense when using the number of $\textrm{SPR}$ moves as a proxy for the number of reticulation events such as hybridisation, recombination, or horizontal gene transfer. Furthermore, $\textrm{HSPR}$ moves provide a wider range of trees at close distance than for example $\textrm{RNNI}$ moves, which is especially useful for tree proposals. These observations demonstrate that studying ranked $\textrm{SPR}$ treespaces may provide insights to better understanding phylogenetic methods for time trees.

We also find some interesting differences between ranked and unranked $\textrm{SPR}$ spaces. One of those is the absence of the (weak and strong) cluster property in ranked $\textrm{SPR}$. This suggests that the work on Maximum Agreement Forests (MAFs) cannot be transferred to ranked $\textrm{SPR}$ spaces (see Whidden and Matsen (2019) for a discussion on MAF-like problems). Since MAFs are essential in the proofs of $\mathcal{N}\mathcal{P}$-hardness for classic $\textrm{SPR}$, a different strategy is needed to prove the complexity of computing distances in $\textrm{RSPR}$ and $\textrm{HSPR}$. Note that it is currently not known whether this problem is $\mathcal{N}\mathcal{P}$-hard in the ranked $\textrm{SPR}$ treespaces.

We did however obtain some results on properties for shortest paths in $\textrm{HSPR}$ and $\textrm{RSPR}$. The order of moves on shortest paths in $\textrm{RSPR}$ can be changed so that first rank moves and then $\textrm{HSPR}$ moves are performed, which suggests that the complexity of computing distances in the two spaces is the same. Furthermore, we found that there is a shortest path on which the ranks of $\textrm{HSPR}$ moves increases monotonically. Leveraging these results in ranked $\textrm{SPR}$ spaces might lead to developing an algorithm for computing or approximating distances in these treespaces. Another characteristic of ranked $\textrm{SPR}$ spaces that makes them stand out from known tree rearrangement based treespaces is that adding one leaf to two trees can decrease their distance linearly in *n*. This is very interesting behaviour, as it has not been observed before in any of the known tree rearrangement based treespaces, and seems to be related to the presence of rogue leaves. It is worthwhile investigating the influence of this effect on tree inference algorithms, especially when considering algorithms that aim to add new sequence data to existing phylogenies (online algorithms).

This paper provides the definition and first analysis of spaces of ranked trees to enable studying time tree inference methods in the same way as untimed tree inference methods. One important open question is that of the complexity of computing distances in $\textrm{RSPR}$ and $\textrm{HSPR}$ treespace. A first step could be to establish that the complexity of computing distances is the same for these two ranked treespaces. Furthermore, the exact diameter of $\textrm{HSPR}$ and $\textrm{RSPR}$ space is still to be determined. We can use our computations (Collienne 2023) to show that the diameter of $\textrm{HSPR}$ follows the formula $\lfloor \frac{3}{2} (n-2)\rfloor$ for $n \le 7$, but whether this is true for any *n* remains an open question. Another very important next step for research on ranked $\textrm{SPR}$ is developing algorithms, ideally fixed-parameter tractable ones, to calculate or approximate distances. This would facilitate leveraging our newly defined treespaces to allow analysing BEAST2 (Bouckaert et al. 2014) output as it has been done for MrBayes output with unranked $\textrm{SPR}$ (Whidden and Matsen 2015, 2017).

## Footnotes

1 https://beast.community/xml_reference#ltfixednodeheightsubtreepruneregraftgt-element.
